# Sex Differences in Reported Effectiveness and Psychosocial Effects of Therapy-Assisted Sexual Orientation Change

**DOI:** 10.7759/cureus.104735

**Published:** 2026-03-05

**Authors:** Donald P Sullins

**Affiliations:** 1 Research, Ruth Institute, Lake Charles, USA; 2 Sociology, Catholic University of America, Washington, DC, USA

**Keywords:** ex-gay, religion, sexual orientation, sexual reorientation therapy, therapy

## Abstract

Background

Sexual reorientation therapy (SRT) refers to voluntary therapeutic interventions for sexual orientation change, which may or may not include a goal of changing same-sex attraction (SSA), behavior, or identification. Research on the contested question of whether SRT is effective or safe consists almost exclusively of clinical studies of males. This study presents two improvements to the evidence: it includes a control group of non-therapy participants and distinguishes outcomes by sex.

Methods

This study was conducted at Ruth Institute, Lake Charles, LA, USA. It examined a cross-sectional nonprobability sample of persons reporting same-sex orientation change (n = 183) for the retrospectively reported extent of reduction in SSA and same-sex behavior (SSB), for exposure to SRT and other forms of therapy, and for positive and negative changes in six psychosocial problem domains: depression, suicidality, self-harm, self-esteem, social function, and alcohol or substance abuse. Reported values were compared by sex (129 men (70.5%) and 54 women (29.5%)) and by SRT exposure (98 (58.7%) were exposed, 69 (41.3%) not exposed, with 16 item nonrespondents). The mean change was assessed by a t-test for continuous variables and Fisher’s exact test for categorical variables.

Results

Thirty-nine (38.6%) of 101 men and 44 (88.0%) of 50 women reported high SSA before changing sexual orientation and then reported low SSA after the change, thereby making a low same-sex attraction (LSSA) shift. SRT exposure increased the percentage of an LSSA shift for women (from three of five (60%) without SRT to 24 of 25 (96%) with SRT, p = 0.06, d = +1.3) but reduced it for men (from 12 of 22 (55%) without SRT to 17 of 44 (39%) with SRT, p = 0.167, d = -0.32). Therapy with a goal of SSA change weakened men’s prospects of an LSSA shift the most (to eight of 26 (31%), p = 0.099, d = -0.49). For both men and women, therapy not directly related to sexual orientation change was most strongly associated with an LSSA shift (17 of 40 men, 30 of 30 women, overall p = 0.029, d = 0.51). Despite the sex difference in reducing SSA, 100% of both men and women reported slight or no SSB after the change (LSSB shift). Psychological problems improved overall, somewhat higher for women (2.84 on a five-point scale) than men (2.50) (p = 0.01, d = 0.38). SRT exposure enhanced overall psychological improvement moderately, by +0.26 (d = 0.34) for women and +0.20 (d = 0.21) for men, but strongly improved outcomes for depression for women (+0.80, p = 0.009, d = 0.69) and self-harm for men (+0.45, p = 0.021, d = 0.39). Previously suicidal persons reported a much stronger benefit from SRT than non-suicidal persons (+0.70, p = 0.002, d = 0.67).

Conclusion

For sexual orientation change, SRT helped women but not men to reduce SSA, which women did to a much greater degree than men. High psychological benefit from change was improved slightly by SRT, more for women than men. Research that excludes women is likely to substantially understate the effectiveness and psychological benefit of SRT. Theoretical, legal, and clinical implications are discussed.

## Introduction

Some highly religious nonheterosexual persons seek therapeutic support to reconcile their deep commitment to religious norms that prohibit homosexual behavior with one or more of the dimensions of sexual orientation, i.e., sexual attraction, behavior, or identification (sexual reorientation therapy (SRT)) [1]. (Note: Appendix A presents for reference a list of all abbreviations mentioned in this study.) SRT is a subset of the activities that the American Psychological Association has labeled “sexual orientation change efforts” (SOCE), which also include non-therapy activities such as study courses or religious retreats. Many research studies confuse or conflate SRT with “conversion therapy,” an outmoded practice from the 1970s, which attempted to reverse homosexual attractions through aversive conditioning [2,3], but SRT is quite distinct: it consists only of talk therapy and may target management of sexual attractions, not change. Conversion therapy clients often attended unwillingly, by court order or family pressure, in a time when homosexuality was illegal and stigmatized, leading to high rates of failure and therapy trauma. SRT clients today participate voluntarily, motivated primarily by religious ideals; none attend under legal coercion, with correspondingly higher rates of success and satisfaction.

Prompted by ideological opposition from professional organizations, both SRT and conversion therapy have been further conflated with therapy for gender identity conflicts, using the acronym “SOGICE” (for “sexual orientation and gender identity change efforts”) [4], in an attempt to ban all three practices in the United States. Efforts to show SRT to be ineffective and psychologically harmful have been based largely on individual anecdotes and recruited convenience samples of LGBT (lesbian, gay, bisexual, or transgender) persons, which thereby exclude, by design, anyone who may have changed sexual identity.

Findings from such efforts have been definitively countered by nationally representative population surveys, which have presented strong evidence of frequent sexual orientation change without increased psychopathology and of an absence of psychological harm from exposure to SRT in the general LGB population. Studies of current heterosexual SRT alumni have also consistently reported a high degree of perceived effectiveness, ranging from modest to complete sexual orientation change, and positive psychological effects from their experience. Although providing helpful evidence on SRT outcomes, such studies to date have been methodologically limited by only including persons who had undergone therapy, effectively lacking a control group to distinguish the effects of therapy from other interventions or efforts related to sexual orientation change. Most have also been susceptible to bias in that the researchers also conducted the therapy in the study or a similar SRT therapy. Studies on both sides of the question of SRT effectiveness and harm have been further limited by a relative neglect of female sexual orientation in favor of an almost exclusive reliance on male experience.

Overlooking women

Even though lesbian and bisexual (LB) women tend to outnumber gay and bisexual (GB) men in population data [5] and women are more likely to change sexual orientation [6], the unique experiences of women have been almost completely neglected in sexual reorientation. Most quantitative studies both opposing and favoring SRT have examined samples that either highly under-represent women [7] or exclude them entirely [3,8-12]. There are no comparable large-scale studies of female samples. A few studies that have employed mixed samples of men and women have not reported differences by sex to any meaningful extent [2,13-18]. Ryan et al. [17] reported that females, who comprised 44.9% of their sample of 245 LGBT young adults, were 1.6 times more likely than males to have experienced parent-initiated SOCE, which was the focus of their study. However, they reported no other information by sex. Chan et al. [13] analyzed a sample of 179 males and 49 females but did not mention any sex differences, despite featuring outcomes, including depression, anxiety, and suicidality, that are known to vary substantially by sex. Green et al. [14] reported the percent cisgender and transgender, but not the percent male and female, in their convenience sample of over 25,000 respondents. Similarly, Nicolosi et al. [16], reporting detailed information on beneficial change in sexual attractions and behavior among 882 currently heterosexual SRT recipients, which included 193 women (22%), made no mention of sex differences, even though Nicolosi’s psychodynamic theory of sexual orientation development proposes that such differences are significant. A notable exception to the inattention to women was Dr. Robert Spitzer’s landmark 2003 study [19] of sexual orientation change, which reported numerous sex differences in sexual attraction and orientation change in a sample similar to that of the present study. Spitzer’s findings are reviewed further in the Discussion section below.

Neglect of women’s experience can substantially bias the assessment of the ineffectiveness and harm of SRT. Males and females have distinct profiles of sexuality and psychological health. Compared to men, women’s sexual attractions and behaviors have been found to be generally more diverse, fluid, flexible, and (as just noted) changeable [6]. Genetic evidence ascribes somewhat higher inheritance to male homosexuality than female [20], and male physiological response to same-sex stimuli appears much less susceptible to change [21]. (Note: In this paper, which interacts with research that uses both sets of terms, the distinction between “homosexual” and “heterosexual” will be used interchangeably with the distinction between “same-sex” and “opposite-sex.”) At the same time, women experience higher rates of internalizing psychological disorders, such as depression and anxiety, and substantially lower rates of suicidality. These differences suggest that extending findings from male samples to all SRT participants may risk understating rates of change and psychological harm, while overstating suicidality.

Study aims and hypotheses

The present study examines the first purposive sample of persons reporting a homosexual to heterosexual sexual orientation change since Spitzer’s landmark 2003 study. Unlike most previous SOCE study samples, this one was not conditioned on sex, current sexual identity, or therapy participation. These data improved upon previous evidence in three ways. First, the data did not censor evidence of sexual orientation change by excluding individuals who currently identified as non-LGBT. Second, the data included a quasi-experimental reference group of persons reporting sexual orientation change who had not undergone therapy. Third, the data included sufficient numbers of both men and women to support meaningful distinctions and comparisons.

The aim of this study is to amend, in small part, the neglect of women’s likely unique characteristics by examining the possibility and extent of differences between men and women in the experience of sexual orientation change. To this end, the study will examine three broad hypotheses regarding sex differences stemming from the prior research reviewed above:

Hypothesis 1 (H1) 

Comparing values prior to sexual orientation change with current values, women more than men, on average, will report more overall reduction in SSA, and SRT will be associated with a greater improvement in SSA reduction.

Hypothesis 2 (H2)

Comparing values prior to sexual orientation change with current values, women more than men, on average, will report more overall reduction in SSB, and SRT will be associated with a greater improvement in SSB reduction.

Hypothesis 3 (H3)

Subtracting reported psychological harm from reported psychological benefit, women more than men, on average, will report a more positive net psychological outcome associated with sexual orientation change, and SRT will be associated with a greater improvement in net psychological outcome.

## Materials and methods

Participants

This study was conducted at the Ruth Institute, Lake Charles, LA, USA. The data for this study consisted of 183 responses to an online survey administered from February 17 to August 31, 2025, to a purposive sample of persons who had desisted from same-sex attraction (SSA), same-sex behavior (SSB), and/or lesbian, gay, or bisexual (LGB) identity. An initial draft of the survey instrument was circulated for comment to 27 subject matter experts representing support groups, counselors, and scholars experienced with persons who had desisted from LGB orientation. Sixteen experts completed the draft survey and/or offered critical comments and suggestions, which functioned as a pre-test to test and validate the survey items. After revision in response to their feedback, the final survey consisted of 84 items, some of which are composite questions with several items. The survey primarily consisted of closed-ended questions with Likertized response sets, with a general open-ended question at the end. The survey items used for this study are described in detail below, and Appendix B presents a copy of the full survey questionnaire. After completing pretest and validation, each subject matter expert was asked to distribute to the persons in their network an invitation email with a link to the final version of the online survey.

The invitation email advised readers that, to qualify for the survey, “you must be 18 or older and have experienced one or more of the following: experienced SSA at some point in the past but now do not do so or do so much less; engaged in same-sex behavior at some point in the past but now do not do so or do so much less; identified as lesbian, gay or bisexual (LGB) at some point in the past but now do not do so” [22]. Prospective participants could click a link in the email to bring up an informed consent screen, which advised of the possible risks of participation and included a link to the survey for those who chose to proceed. The screen advised participants that the survey would ask detailed questions about their personal sexual attractions and behavior, but it was completely anonymous, and any question could be skipped. The study administration procedures and informed consent text were reviewed and approved by the Catholic University of America Institutional Review Board (Certificate 24-0091, issued December 2, 2024). Copies of the invitation email and informed consent screen are included in the supplemental material.

The online survey consisted of 84 items, some of them composite, and took an average of 21.8 minutes to complete. English-speaking participants from 30 countries responded to the survey. To preserve confidentiality, specific country information has been suppressed.

After correctly answering the screening questions, 187 eligible persons responded to the survey. Technical protocols suppressed multiple survey attempts, and pattern screening for malicious responses or jokesterism was negative. Subsequent data cleaning removed four cases: one that did not meet the screening criteria and three with irreconcilable logically inconsistent responses. Of the remaining 183 cases, 155 completed the entire survey, but an additional 28 began the survey but dropped out before reaching the end. Survey metrics permitted us to distinguish between persons who never saw a question because they had dropped out (completion nonresponse) and persons who saw the question but declined to answer it (item nonresponse). We determined the analytical sample to be the total number of persons who saw each question. The total number of cases (n) for analysis thus ranged from 155 to 183, depending on the question. For some questions, the usable number of cases was reduced further due to item nonresponse. Further sampling detail is provided in the “Question Pro LBP Data Codebook Report” in the supplemental material. The analysis below makes use of all available cases for each question. 

Measures

Measures of sexual attractions and behavior were derived from the Sell Assessment of Sexual Orientation [23]. Like the more common Kinsey scale, the Sell Assessment employs Likertized response sets that facilitate precise measurement of incremental intraindividual changes and small differences between individuals. In norming with a sample of 177 men, the Sell Assessment demonstrated 93% test-retest reliability and 85% construct validity with the Kinsey scale [24]. However, unlike the Kinsey scale, which imposes the counterfactual assumption that SSA and opposite-sex attraction are on a spectrum so that more SSA necessarily means less OSA, the Sell Assessment permits respondents to report OSA independently from SSA. Due to its ability to distinguish sexual identity (a social construct) from behavior and/or attraction (personal characteristics), the Sell Assessment has been widely used in medical research.

Sexual attraction was measured by questions asking “the most you were sexually attracted to a man” and “the most you were sexually attracted to a woman” for three different time periods: during the past year before taking the survey, during “the period when you experienced the greatest same-sex attraction,” and ever in the respondent’s life. The response options, coded 1-7, were “not at all sexually attracted”, “slightly sexually attracted”, “mildly sexually attracted”, “moderately sexually attracted”, “significantly sexually attracted”, “very sexually attracted”, and “extremely sexually attracted”. Measures of SSA and opposite-sex attraction (OSA) at each time period were constructed by matching the answers to these questions with the respondent’s sex. For analysis, the three highest options (significantly, very, and extremely) were classified as “high SSA” and the three lowest options (not at all, slightly, and mildly) as “low SSA.” Persons with high SSA before change and low SSA after change were classified as having made a “low SSA shift” (LSSA shift).

A similar set of questions asked how often the respondent had sexual contact with a man and with a woman during the same three time frames. The response options, coded 1-7, were “never”, “less than 1 time a month”, “1-3 times a month”, “1 time a week”, “2-3 times a week”, “4-6 times a week”, and “daily”. From these responses were constructed measures of SSB and opposite-sex behavior (OSB). For the analysis, the three highest options (two to three times a week, four to six times a week, and daily) were classified as “high SSB” and the three lowest options (never, less than one time a month, and one to three times a month) as “low SSB.” Persons with high SSB before change and low SSB after change were classified as having made a “low SSB shift” (LSSB shift).

In a similar way, change effects were observed by comparing values reported for the past year with those reported for the period that the respondent experienced the greatest SSA or SSB or least OSA or OSB. The former are denoted in this study as “current” values after change; the latter as “prior” values before any change. For most measures of change, change was assessed by subtracting the current values after change from the prior values before change. In this way, sexual orientation change was not directly reported or characterized by the respondents but was independently computed by the researcher.

Measures of perceived psychosocial harm and benefit were adapted from Spitzer’s [19] seminal study of SOCE participants, which were in turn based on the themes developed in earlier studies. Very similar measures using the same domain typology have also been employed in two more recent studies of SOCE clients [8,12]. Replicating these metrics facilitates construct validation and comparison of findings with previous research. To assess perceived psychosocial changes, a series of items asked respondents: “Looking back on your personal change journey, how much positive [negative] change (symptoms changing for the better [worse]) would you say you have experienced in each of the following areas?” The specified domains were 1) depression, 2) thoughts/attempts of suicide, 3) alcohol and substance abuse, 4) self-harmful behavior, 5) social functioning, and 6) self-esteem. Response options were “none”, “slight”, “moderate”, “very much”, and “extremely much”, numbered 1-5, with 6 indicating “not applicable.”

The “not applicable” response count varied widely, ranging from 1 to 83 across the six domains, and was not the same for positive and negative changes for the same domain. To facilitate comparative analysis, “not applicable” responses were set to the item mean, with the remaining five options coded 0-4. The measure of perceived negative change was then subtracted from perceived positive change to produce a single statistic indicating net change for each area, which could be positive or negative. Changes, i.e., positive, negative, and net, were then averaged over all six domains to produce measures of perceived overall psychosocial benefit (improvement of symptoms), harm (worsening of symptoms), and net harm/benefit.

Therapy experience was measured by six items in which respondents indicated if they had ever seen a professional counselor or therapist for various reasons (followed by number and percent indicating yes): A) “to help you change your sexual identity away from homosexuality and/or to/toward heterosexuality?” (54, 30%); B) “to help you reduce same-sex behavior and/or increase opposite-sex behavior?” (49, 27%); C) “to help you reduce same-sex attractions and/or increase opposite-sex attractions?” (52, 28%); D) “to address discomfort or struggle with issues related to your sexuality without expecting or excluding change?” (56, 31%); E) “to help you remain or become more comfortable with a homosexual sexual orientation?” (8, 4%); or F) “for any other reason” (72, 39%). A total of 131 respondents (78%), comprised of 85 men (66%) and 46 women (85%), indicated that they had seen one or more counselors or therapists; 76 (42% of all respondents or 58% of those exposed to therapy) had seen more than one type, seeing on average 2.2 counselors. A total of 98 respondents (54%), 68 men (53%) and 30 women (56%), had gone to some form of SRT: 71 (39%) to “change SRT” (response options A, B, and C) and 56 (34%) to “non-change SRT” (response option D). Response option E could not be meaningfully combined with another category and contained too few responses (eight, with only two women) to analyze independently. Response option F (“non-SRT therapy”) was selected by 72 respondents (39%), 41 men (32%) and 31 women (57%), who indicated seeing one or more counselors or therapists for a wide range of psychological issues not directly related to sexual orientation change.

Suicide ideation was measured by a question which asked, “Have you ever seriously considered attempting suicide?” with possible responses of “No,” “Just once,” or “More than once.” For analysis, the latter two responses were combined to form a dichotomous variable reporting whether or not the respondent had ever seriously considered attempting suicide.

Analyses

Pertinent effects in the data were analyzed under the theoretical paradigm of classic elaboration analysis [25], which examines and elaborates direct and indirect mean differences in survey data. Bivariate contrasts were assessed for significance and magnitude using the t-test, Cohen’s d statistics, and Fisher’s exact test. To ensure sufficient conformity with distributional assumptions for the first two measures (normality and interval scale), self-reported differences before and after SOCE were also initially assessed by a two-tailed signed test, which does not assume these characteristics of the data. The two tests reported closely similar results with no differences in hypothesis assessment, thereby assuring the practical accuracy of the t-test results in these data.

Following convention, findings with a p-value of 0.05 or less were considered statistically significant in this study. Findings with a p-value of 0.10 or less but greater than 0.05, which would be statistically significant in an analysis that used the 0.10 critical standard, may be identified as effects of interest due to their marginal strength but were not considered to have met the conventional standard for statistical significance. Significance may be interpreted, along with effect size, as at a minimum a functional indicator of the strength of association. Exact p-values are reported for all results, which permits the reader to interpret statistical significance by a different standard if desired. Statistical significance is an uncertain metric for any non-random sample. In this study, statistical significance may indicate, at best, that results may be generalized to the hypothetical population of persons likely to respond to similar sampling procedures, which in turn may reflect the general population of persons who perceive themselves to have changed one or more aspects of sexual orientation.

Effect sizes, expressed as Cohen’s d, reported differences in terms of standard deviation and thus provided an indication of the magnitude of change or contrast that was comparable across differently scaled variables. The substantive interpretation of effect sizes is a matter of some disagreement and varies according to the variables being considered; however, using benchmarks originally suggested by Cohen [26], a value below 0.3 is generally interpreted as small, 0.3-0.6 moderate, and above 0.6 as indicating a large effect. Analyses for the present study made use of IBM SPSS Statistics for Windows, Version 25.0 (released 2017, IBM Corp., Armonk, NY) and Stata Statistical Software (release 18, StataCorp LLC, College Station, TX). The survey was hosted by QuestionPro (www.questionpro.com).

## Results

Sample characteristics

Sample respondents consisted of disproportionately White, older Christian individuals who were highly educated and highly religious. The mean age was 49.7 years (standard deviation (SD) 14.0), with women (54.8 years, SD 12.2, n = 52) significantly older than men (47.7 years, SD 14.2, n = 127; t = -3.17, p = 0.002) by 7.1 years, on average. Table 1 presents other sample demographic characteristics by sex. The majority of sample members (70%) held a college degree; a third (32%) held a postgraduate degree. Almost all (90%) reported church attendance at least weekly, and over half (52%) more than once a week. Protestants (59%) outnumbered Catholics (33%) among the 91% of Christians in the sample. Most respondents (55%) had never been married, although a sizable minority (37%) were currently married to someone of the opposite sex.

**Table 1:** Selected sample demographic characteristics by sex (N = 183). Equality tested by Fisher's exact test. Number of cases was reduced for some variables due to item non-response. n, number of cases; M, mean; SD, standard deviation

Variable	N	%	Men (n = 129)		Women (n = 54)		Fisher’s exact test: men = women
			N	%	N	%	P
Ethnicity							
White	151	82.5	106	82.2	45	83.3	0.476
Black	9	4.9	6	4.7	3	5.6	
Hispanic	6	3.3	6	4.7	0	0	
Asian	15	8.2	10	7.8	5	9.3	
Other	2	1.1	1	0.8	1	1.9	
Sexual identity							
Heterosexual	92	50.8	57	44.9	35	64.8	0.029
Gay or lesbian	11	6.1	9	7.1	2	3.7	
Bisexual	17	9.4	16	12.6	1	1.9	
Other	61	33.7	45	35.4	16	29.6	
Highest education/degree							0.865
High school	14	7.7	11	8.5	3	5.6	
Some college/associate’s	41	22.4	30	23.3	11	20.4	
Bachelor’s	69	37.7	47	36.4	22	40.7	
Master’s degree	40	21.9	29	22.5	11	20.4	
Doctoral or professional	19	10.4	12	9.3	7	13.0	
Religious affiliation							0.007
Protestant	107	58.5	67	51.9	40	74.1	
Catholic	60	32.8	51	39.5	9	16.7	
Other	16	8.8	11	8.5	5	9.3	
Religious service attendance							0.625
Daily	14	7.7	12	9.3	2	7.7	
More than once a week	81	44.3	56	43.4	25	44.3	
Once a week	69	37.7	46	35.7	23	42.6	
Once or twice a month	12	6.6	9	7.0	3	5.6	
A few times a year	3	1.6	2	1.6	1	1.9	
Seldom or never	4	2.2	4	3.1	0	0	
Current marital status							0.151
Never married	100	54.6	67	51.9	33	61.1	
Married, opposite-sex	68	37.2	53	41.1	15	27.8	
Married, same-sex	3	1.6	3	2.3	0	0	
Divorced	9	4.9	4	3.1	5	5	
Widowed, separated, cohabiting	3	1.6	2	1.6	1	1	
World region							0.298
North America	114	62.3	80	62.0	34	63.0	
Europe	37	20.2	29	22.5	8	14.8	
Asia and Oceania	28	15.3	16	12.4	12	22.2	
Other	4	2.2	4	3.1	0	0	

Most respondents (62%) were from the USA and Canada, with another 20% from Europe, 8% from Asia, and 8% from Oceania. As noted, anonymity safeguards precluded more precise location disclosure, and the survey was administered only in English.

Of the 183 respondents, 129 (70.5%) identified as male and 54 (29.5%) as female. Women averaged about 7.5 years older than men, and were more likely to report Protestant rather than Catholic religious affiliation and to currently identify as heterosexual. Women in the sample were also less likely than men to be married. Otherwise, demographic differences between the two groups were small and not statistically significant.

Continuous change measures

Table 2 presents the means and standard deviations by sex for the continuous measures of sexual attraction and behavior in the study. The results preview more detailed findings presented below. “Prior” refers to recalled values before the target sexual orientation change. “Current” reports current values after change. Both men and women reported a similar profile of prior to current changes: large reductions in SSA and SSB paired with smaller increases in OSA and OSB. Women, however, experienced more extreme changes than men: as a group, they reported larger reductions in SSA and SSB, and smaller increases in OSA and OSB, than did men.

**Table 2:** Self-reported sexual attraction and behavior prior to change and currently after change, by sex. Prior, previous value prior to sexual orientation change; Current, current value after sexual orientation change; N, number of cases; SD, standard deviation; SSA, same sex attraction; OSA, opposite sex attraction; SSB, same sex behavior; OSB, opposite sex behavior; T, t statistic; P, p-value for statistical significance of t statistic; d, Cohen’s d

Variable	Prior (before change)			Current (after change)			Change (Current - Prior)			T-test: Current = Prior		Effect size
	N	Mean	SD	N	Mean	SD	N	Mean	SD	T	P	d
Men only												
SSA	115	6.03	1.35	116	3.82	1.94	115	-2.19	1.78	13.2	.000	1.32
SSB	125	3.40	1.84	126	1.27	0.59	124	-2.13	1.90	12.5	.000	1.57
OSA	113	2.15	1.65	113	3.55	2.00	111	1.46	1.66	-9.29	.000	-.76
OSB	114	1.33	0.93	123	1.92	1.45	112	0.49	1.56	-3.33	.001	-.48
Women only												
SSA	53	6.08	0.90	53	1.85	1.39	53	-4.23	1.51	20.3	.000	3.61
SSB	54	4.48	1.83	54	1.02	0.14	54	-3.46	1.83	13.9	.000	2.67
OSA	53	2.13	1.44	52	2.90	1.90	52	0.75	1.77	3.06	.004	-.46
OSB	51	1.27	0.85	52	1.69	1.16	50	0.44	1.43	2.17	.035	-.44
All cases												
SSA	168	6.04	1.22	169	3.21	2.00	168	-2.83	1.94	18.9	.000	1.71
SSB	179	3.73	1.90	180	1.19	0.51	178	-2.53	1.97	17.1	.000	1.83
OSA	165	2.14	1.58	165	3.35	1.99	163	1.23	1.72	-9.15	.000	-.67
OSB	165	1.32	0.90	175	1.85	1.37	162	0.48	1.52	-3.99	.000	-.46

Prior to change, both men and women reported the same or very similar: mean SSA (t = 0.24, p = 0.808) at just above 6.0 (consistent with “very sexually attracted”); mean OSA (t = 0.07, p = 0.945) at just above 2.1 (“slightly sexually attracted”); and mean OSB frequency (t = 0.39, p = 0.700) at about 1.3 (between “never” and “less than 1 time a month”). Women reported significantly higher prior mean SSB frequency than men (t = 3.62, p = 0.000), at 4.5 (between “1 time a week” and “2-3 times a week”) compared to 3.4 (between “1-3 times a month” to “1 time a week”) for men.

Both men and women strongly reduced same-sex attraction and behavior and increased opposite-sex attraction and behavior, with statistically significant effect sizes ranging from moderate to very large. After the change, however, women reported lower current mean values for all four measures of attraction and behavior. As indicated by the effect sizes, women’s pre- to post-change decline in SSA was twice as large as that of men (3.61 compared to 1.32). The excess of women’s over men’s decline in SSB frequency was a little smaller (2.67 compared to 1.57) but may be more telling, since women dropped to a current SSB of 1.0 (“never”), the lowest level possible. By contrast, reported OSB increased only modestly over the change journey for both men and women (with effect sizes of 0.44 to 0.46) and reported OSA increased more for men than for women (0.76 to 0.46). Table 3 presents direct tests of the sex differences for sexual orientation change, confirming that change in SSA and SSB were significantly higher for women, change in OSA significantly lower, and there was no sex difference in change in OSB, which was comparatively small for both men and women.

**Table 3:** Sex differences in reported sexual orientation change N, number of cases; SD, standard deviation; SSA, same sex attraction; OSA, opposite sex attraction; SSB, same sex behavior; OSB, opposite sex behavior; T, t statistic; P, p-value for statistical significance of t statistic; d, Cohen’s d

Variable	All cases			Men only			Women only			T-test: Men = Women		Effect size
	N	Mean	SD	N	Mean	SD	N	Mean	SD	T	P	d
SSA change	168	-2.83	1.94	115	-2.19	1.78	53	-4.23	1.51	7.22	.000	1.20
SSB change	178	-2.53	1.97	124	-2.13	1.90	54	-3.46	1.83	4.34	.000	.71
OSA change	163	1.23	1.72	111	1.46	1.66	52	0.75	1.77	-2.49	.014	-.42
OSB change	162	0.48	1.52	112	0.49	1.56	50	0.44	1.43	-.197	.844	-.03

Shifts in sexual attraction and behavior

**Figure 1:**
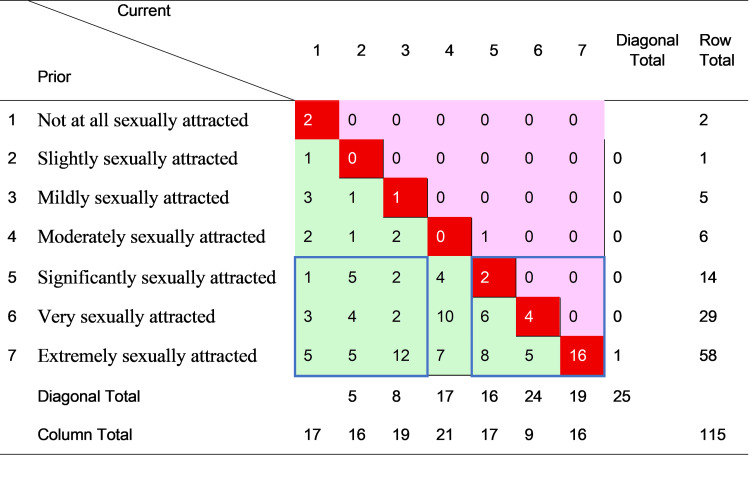
Cross-tabulation of prior and current sexual attraction to men–male cases only (n = 115) The data are presented as case counts. Cells in bright red (the main diagonal) signify no change; cells in pink (upper off-diagonal) signify change toward greater homosexual attraction; cells in green (lower off-diagonal) signify change toward greater heterosexual attraction. “Diagonal total” reports the sum of the corresponding diagonal group of cells, parallel to the main diagonal. N, number of cases; current, current values after change; prior, previous values before change. The item prompted, “The most you were sexually attracted to a man was….” Image created by the author with MS Excel (Microsoft Corp., USA)

**Figure 2:**
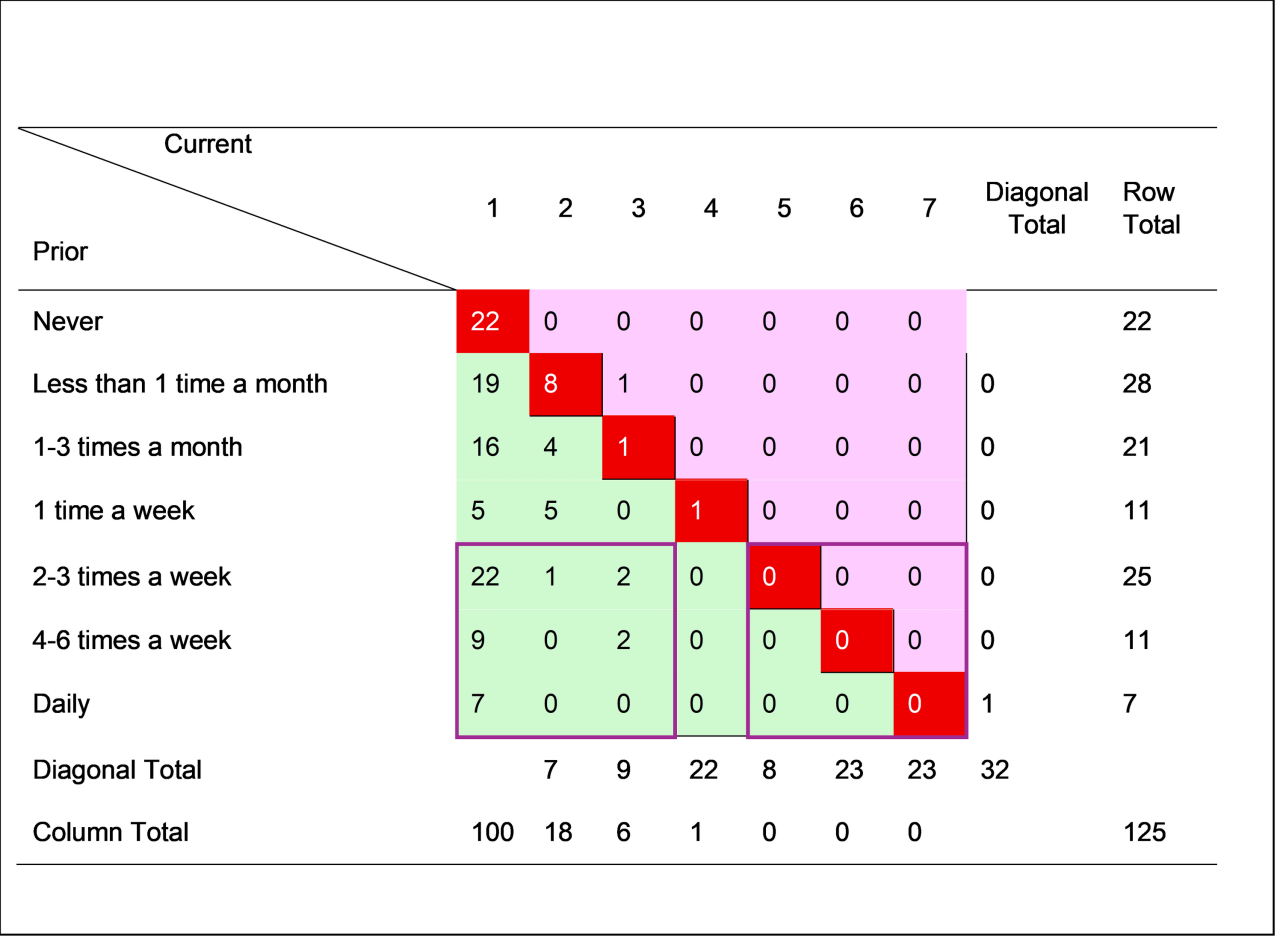
Cross-tabulation of prior and current sexual contact frequency with men–male cases only (n = 125) The data are presented as case counts. Cells in bright red (the main diagonal) signify no change; cells in pink (upper off-diagonal) signify change toward greater sexual contact frequency; cells in green (lower off-diagonal) signify change toward less sexual contact frequency. “Diagonal total” reports the sum of the corresponding diagonal group of cells, parallel to the main diagonal. N, number of cases current, current values after change; prior, previous values before change. The question was, “How often did you have sexual contact with a man?” Image created by the author with MS Excel (Microsoft Corp., USA)

**Figure 3:**
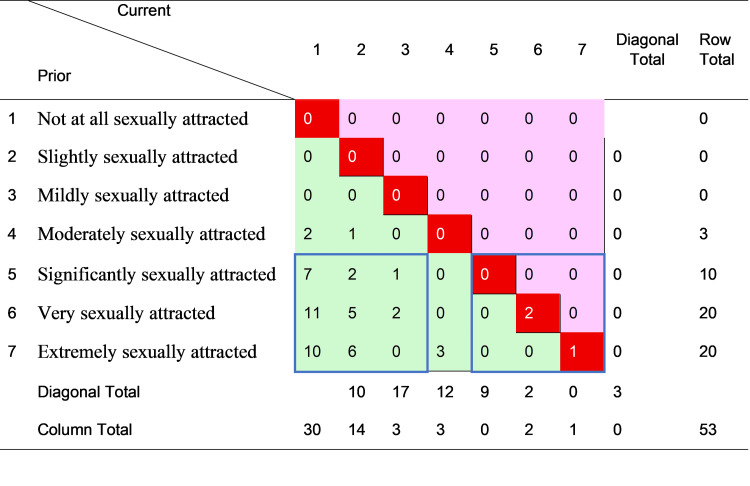
Cross-tabulation of prior and current sexual attraction to women–female cases only (n = 53) The data are presented as case counts. Cells in bright red (the main diagonal) signify no change; cells in pink (upper off-diagonal) signify change toward greater homosexual attraction; cells in green (lower off-diagonal) signify change toward greater heterosexual attraction. “Diagonal total” reports the sum of the corresponding diagonal group of cells, parallel to the main diagonal. N, number of cases current, current values after change; prior, previous values before change. The item prompted, “The most you were sexually attracted to a woman was….” Image created by the author with MS Excel (Microsoft Corp., USA)

**Figure 4:**
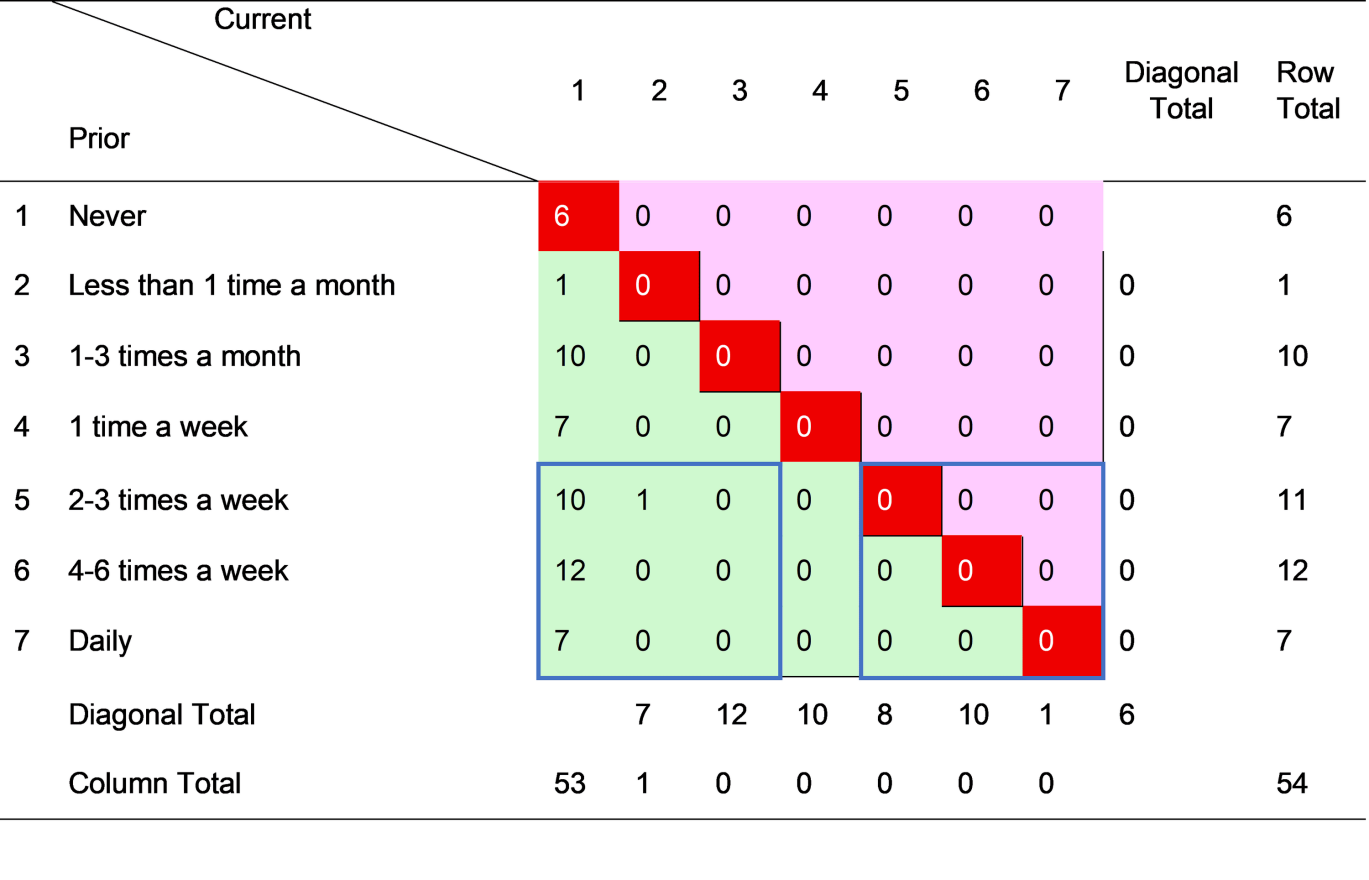
Cross-tabulation of prior and current sexual contact frequency with women–female cases only (n = 54) The data are presented as case counts. Cells in bright red (the main diagonal) signify no change; cells in pink (upper off-diagonal) signify change toward greater sexual contact frequency; cells in green (lower off-diagonal) signify change toward less sexual contact frequency. “Diagonal total” reports the sum of the corresponding diagonal group of cells, parallel to the main diagonal. N, number of cases; current, current values after change; prior, previous values before change. The question was, “How often did you have sexual contact with a woman?” Image created by the author with MS Excel (Microsoft Corp., USA)

Figures 1-4 cross-tabulate reported prior and current sexual attraction and behavior for men and women, respectively, forming mobility tables which enable direct observation of the extent of change in self-reported attraction and behavior over the LPB (“leaving pride behind”) transition. For each figure, the cells in the main diagonal (shaded red) represent cases where current sexual attraction or behavior was the same as it was prior to change. For these individuals, the change journey did not involve any transition in sexual attraction or behavior. The remainder of the cells in the table represent cases where reported sexual attraction or behavior was currently different than before the change. The cells to the upper right of the diagonal (shaded pink) show cases where before-to-after sexual attraction or behavior moved toward greater same-sex orientation. The cells to the lower left of the diagonal (shaded green) show cases where sexual attraction or behavior moved toward less same-sex orientation.

Inspection of Figure 1 reveals that of the 115 males who disclosed their level of SSA before and after the LPB transition, one (0.9%) moved toward greater SSA, 25 (21.7%) experienced no change, and 89 (77.4%) moved toward lower SSA. Similarly, of the 125 males who reported their prior and current SSB shown in Figure 2, one (0.8%) moved toward more frequent SSB, 32 (25.6%) reported no change, and 92 (73.6%) moved toward less SSB. Notably, most (16 of 25, or 64%) of the males with stable SSA in Figure 1 reported the highest level of SSA, i.e., “extremely sexually attracted” to men, whereas most (22 of 32, of 69%) of the males with stable SSB in Figure 2 reported the lowest level of SSB, i.e., “never” engaging in SSB.

By the same analysis, women were more likely to change, and only in a less same-sex direction. Of the 53 females reporting SSA before and after the change in Figure 3, only three (6%) experienced no change, and only six (11%) of the 54 females did not change SSB. Thus, 94% (50 of 53) of women moved toward lower SSA, and 89% (48 of 54) toward lower SSB, over their LPB transitions. The relative absence of movement toward greater same-sex orientation, for both men and women, reflects sample selection, which screened in persons who had desisted from or resisted formerly higher homosexual attraction, behavior, or identity. Although it is hypothetically possible for someone to change in one dimension of sexuality in a different direction than other dimensions, these results indicate that such incongruence was rare in those who had transitioned away from same-sex attraction, behavior, or identity.

To examine sex differences more clearly, the arrays in Figures 1-4 were partitioned into groups of cases representing definite areas of change and stability. The bottom three rows in each figure correspond to individuals who, prior to the LPB transition, reported the highest levels of SSA or the most frequent SSB. These persons had the strongest potential for reduction in SSA and/or SSB. Ignoring the indeterminate cases in the middle, this bottom band was divided into quadrants of three columns each on the right and the left. These two groups of nine cells are indicated on each figure by a blue border. The bottom right quadrant thus represents persons who reported the highest levels of attraction or behavior both before and after transition, that is, those with relatively unchanging attractions and/or behavior (“stable” group). The cases in the bottom left quadrant, on the other hand, indicate those who reported the highest levels of attraction and behavior prior to transition but the lowest levels after transition, making a definite shift from high to low same-sex attraction or behavior (“LSSA (low same-sex attraction) shift” or “LSSB (low same-sex behavior) shift” groups).

Table 4 summarizes the analysis, comparing the magnitude of the stable and LSSA/LSSB shift groups in all four figures. The results were very different by sexual orientation dimension (SSA or SSB) and by sex (men or women). For SSA, the large majority of both men (88%) and women (94%) reported high levels prior to the LPB transition. However, men were much less likely than women to undergo an LSSA shift (39% to 88%) and more likely to remain stable with high SSA (41% to 6%), with the result that after transition, 37% of men, compared to only 4% of women, still reported high SSA. The sex difference in reducing SSA, however, did not extend to a similar difference in reducing SSB. Notably, all (100%) of both men and women reported making an LSSB shift, with the definitive result that no sample respondent of either sex reported a high level of SSB after transition. This striking outcome was not a product of sample selection, nor was it observed for SSA (or sexual identity, not shown).

**Table 4:** SSA and SSB change and stability, by sex, showing effects in Figures 1-4 “High level” denotes values of 5, 6, or 7 (the three highest values) on the seven-point response scale of sexual attraction or behavior. For stable, moderate change, and LSSA or LSSB shift, percents report the percentage of cases at prior high level. SSA, same-sex attraction; SSB, same-sex behavior; n, number of cases; %, percent; LSSA, low same-sex attraction; LSSB, low same-sex behavior; Prior, values prior to change; Current, current values after change

	Men		Women		Total Cases	
SSA	n	%	n	%	n	%
Total reporting	115 (Fig. 1)		53 (Fig. 3)		168	
Prior high level (%)	101	87.8%	50	94.3%	151	89.9%
Stable (%)	41	40.6%	3	6.0%	44	29.1%
Moderate change (%)	21	20.8%	3	6.0%	24	15.9%
LSSA Shift (%)	39	38.6%	44	88.0%	83	55.0%
Current high level (%)	42	36.5%	2	3.8%	44	26.2%
SSB						
Total reporting	125 (Fig. 2)		54 (Fig. 4)		179	
Prior high level (%)	43	34.4%	30	55.6%	73	40.8%
Stable (%)	0	0%	0	0%	0	0%
Moderate change (%)	0	0%	0	0%	0	0%
LSSB Shift (%)	43	100%	30	100%	73	100%
Current high level (%)	0	0%	0	0%	0	0%

Effectiveness of therapy

The unexpected finding that every respondent reported having made an LSSB shift precluded examining the association of SRT with this outcome, but the same was not true for SSA. Did therapy help or alternatively hinder making an LSSA shift? The answer is highly differentiated by sex and by the type of therapy involved. Tables 5 and 6 present the data.

**Table 5:** Low same-sex attraction (LSSA) shift by therapy participation. “Therapy > No Therapy” tests the hypothesis that an LSSA shift was more frequent with therapy than with no therapy. The table includes only 126 cases with high SSA prior to change which either shifted or remained stable. This is one less than reported in Table 4 because one person did not report therapy participation. Therapies were not mutually exclusive; respondents could report multiple therapies. Reference group for all therapies is those exposed to no therapy at all. LSSA shift, low same-sex attraction shift; N, number of cases; %, percent; P, p-value for Fisher’s exact test (one-sided); d, standardized effect size for comparing group means; SRT, sexual reorientation therapy

	Therapy		No therapy		Fisher’s exact test: therapy > no therapy	Effect size
	LSSA shift		LSSA shift			
	No	Yes	No	Yes		
Type of therapy	N (%)	N (%)	N (%)	N (%)	P	
Men (n = 80)						
Any SRT	27 (61.4%)	17 (38.6%)	10 (45.5%)	12 (54.5%)	.167	-.32
Change SRT	23 (65.7%)	12 (34.3%)	10 (45.5%)	12 (54.5%)	.109	-.41
SSA change	18 (69.2%)	8 (30.8%)	10 (45.5%)	12 (54.5%)	.085	-.49
SSB change	17 (65.4%)	9 (34.6%)	10 (45.5%)	12 (54.5%)	.137	-.40
Non-change SRT	17 (63.0%)	10 (37.0%)	10 (45.5%)	12 (54.5%)	.174	-.35
Non-SRT therapy	13 (43.3%)	17 (56.7%)	10 (45.5%)	12 (54.5%)	.551	.04
Any therapy	31 (53.5%)	27 (46.6%)	10 (45.5%)	12 (54.5%)	.349	-.16
Women (n = 46)						
Any SRT	1 (4.0%)	24 (96.0%)	2 (40.0%)	3 (60.0%)	.064	1.30
Change SRT	0 (0%)	13 (100%)	2 (40.0%)	3 (60.0%)	.065	1.46
SSA change	0 (0%)	11 (100%)	2 (40.0%)	3 (60.0%)	.083	1.37
SSB change	0 (0%)	7 (100%)	2 (40.0%)	3 (60.0%)	.152	1.15
Non-change SRT	1 (7.1%)	13 (92.9%)	2 (40.0%)	3 (60.0%)	.155	.93
Non-SRT therapy	0 (0%)	30 (100%)	2 (40.0%)	3 (60.0%)	.017	2.10
Any therapy	1 (6.5%)	40 (93.5%)	2 (40.0%)	3 (60.0%)	.028	1.69
All cases (n = 126)						
Any SRT	28 (40.6%)	41 (59.4%)	12 (44.4%)	15 (55.6%)	.452	.08
Change SRT	23 (47.9%)	25 (52.1%)	12 (44.4%)	15 (55.6%)	.481	-.07
SSA change therapy	18 (48.7%)	19 (51.4%)	12 (44.4%)	15 (55.6%)	.469	-.08
SSB change therapy	17 (51.5%)	16 (48.5%)	12 (44.4%)	15 (55.6%)	.388	-.14
Non-change SRT	18 (43.9%)	23 (56.1%)	12 (44.4%)	15 (55.6%)	.581	.01
Non-SRT therapy	13 (21.7%)	47 (78.3%)	12 (44.4%)	15 (55.6%)	.029	.51
Any therapy	32 (34.9%)	67 (65.1%)	12 (44.4%)	15 (55.6%)	.172	.25

**Table 6:** Sex differences in low same-sex attraction (LSSA) shift by therapy participation (n = 126) “Women > Men” tests the hypothesis that an LSSA shift was more frequent for women than for men. Therapy types were not mutually exclusive; respondents could report participating in multiple types of therapy. The table includes only 126 cases with high SSA prior to change, which either shifted or remained stable currently following change. This case count is reduced by one from that reported in Table 3 due to one case of non-response for therapy participation. LSSA shift, low same-sex attraction shift; N, number of cases; %, percent; P, p-value for Fisher’s exact test (one-sided); d, standardized effect size for comparing group means; SRT, sexual reorientation therapy; SSA, same-sex attraction

	Men		Women		Test: women > men	Effect size
	LSSA shift		LSSA shift			
	No	Yes	No	Yes		
Type of therapy	N (%)	N (%)	N (%)	N (%)	P	Cohen’s d
No therapy	10 (45.5%)	12 (54.5%)	2 (40.0%)	3 (60.0%)	0.612	0.11
Any therapy	31 (53.5%)	27 (46.6%)	1 (2.4%)	40 (97.6%)	0.000	1.28
SRT	27 (61.4%)	17 (38.6%)	1 (4.0%)	24 (96.0%)	0.000	1.39
Change SRT	23 (65.7%)	12 (34.3%)	0 (0%)	13 (100%)	0.000	1.59
SSA change	18 (69.2%)	8 (30.8%)	0 (0%)	11 (100%)	0.000	1.74
SSB change	17 (65.4%)	9 (34.6%)	0 (0%)	7 (100%)	0.003	1.50
Non-change SRT	17 (63.0%)	10 (37.0%)	1 (7.1%)	13 (92.9%)	0.001	1.30
Non-SRT therapy	13 (43.3%)	17 (56.7%)	0 (0%)	30 (100%)	0.000	1.22

Table 5 suggests that, compared to men, women had much higher rates of making an LSSA shift and benefited more from therapy in doing so. Participation in any form of SRT, both change and non-change, had a negative association with an LSSA shift for men but was positively associated with an LSSA shift for women. The difference was large and definitive. For women, the association of an LSSA shift with SRT was highly significant, with a large effect size (+1.3). For men, the association was not significant, with a negative effect size (-0.32), indicating that men were more likely to report undergoing an LSSA shift without therapy than with it. Over half (55%) of men not exposed to SRT reported making an LSSA shift, but only 39% of those undergoing SRT. By contrast, women not exposed to SRT made an LSSA shift at about the same rate as men (60%), but with SRT, almost all women (96%) made an LSSA shift. One-sixth fewer men (16%) but over a third (36%) more women reported an LSSA shift with SRT than did so without it.

Ironically, therapy focused on SSA change had the strongest negative association of any type of therapy with men’s prospects for an SSA shift. Less than a third of men (31%) undergoing therapy for SSA change reported making an LSSA shift, compared to 55% of men without any therapy. This difference approached significance (p = 0.85), with the strongest negative effect size against an LSSA shift for men (d = -0.49).

Table 6 presents formal tests of the sex differences in the association of therapy with reducing SSA reported in Table 5. In these results, men and women unexposed to therapy had effectively the same rates (p = 0.612) of undergoing an LSSA shift over the change transition. With any form of therapy, however, the percentage of women undergoing an LSSA shift greatly exceeded that of men. Every sex contrast was highly statistically significant, at 0.003 or less, with very large effect sizes, ranging from 1.22 upwards. The sex effect for change SRT (1.59) was larger than for non-change SRT (1.30). The largest sex difference (1.74) was for SRT focused on SSA change, confirming (as already observed in Table 5) that therapy directly oriented to SSA change was associated with the least amount of LSSA shift for men relative to women. 

The association of therapy with the mean change in SSA (not shown), which captures smaller partial changes, confirmed these results. Mean SSA reduction for men with change SRT was -1.91 (SD 1.61), -1.97 (SD 1.63) with non-change SRT, but -2.10 (SD 1.80) without any therapy. Women experienced a mean SSA reduction of -4.53 (SD 1.25) with change SRT, -3.88 (SD 1.54) with non-change SRT, and -3.17 (SD 2.64) without any therapy. As with the LSSA shift, women reported much greater mean SSA change than men, which was improved by SRT, especially SRT focused on change, while men reported less mean SSA change, which was lower with SRT, especially SRT focused on change.

Surprisingly, the most helpful type of therapy for an SSA shift was not any of the targeted change therapies or non-change-oriented therapy for sexuality struggles, but therapy that addressed psychological issues not necessarily or directly related to sexuality, such as depression, anxiety, or substance abuse. For women, exposure to such therapy had a large, positive, and significant effect (d = 2.1), the strongest driver of their overall better therapy outcomes. For men, although the effect was small and not statistically significant, therapy for non-sexual psychological issues was the only type of therapy whose estimates were not negatively associated with achieving an SSA shift. 

When not elaborated by sex, the overall results in this sample, in which men outnumbered women, tended to mask the contrasting effects by sex of SRT on the prospect of an LSSA shift. Over all cases, 59% of respondents who had undergone SRT reported making an LSSA shift, compared to 56% of non-SRT participants. Without examining sex differences, this could be incorrectly interpreted to show that therapy was of little effect on SSA change for both men and women, illustrating the importance of considering sex differentials when assessing the effect of SRT.

Psychological harm or benefit

Table 7 presents values from a series of questions that asked respondents for separate ratings for both positive change (how much their mental health symptoms got better) and negative change (how much their mental health symptoms got worse) following sexual orientation change in six psychological problem domains, i.e., depression, suicide, substance abuse, self-harm, social functioning, and self-esteem, and for all domains combined. The response options were a five-point Likertized scale ranging from none (0) to extremely much (4). Values for worse were subtracted from values for better to produce a measure of reported net psychological change.

**Table 7:** Reported change (for better and for worse) in psychological symptoms after sexual orientation change Net column reports Better minus Worse. T-tests tested the hypotheses that women’s positive values were greater than men’s, women’s negative values were less than men’s, and net values were greater than men’s. N, number of cases; SD, standard deviation; SSA, same sex attraction; OSA, opposite sex attraction; SSB, same sex behavior; OSB, opposite sex behavior; P, p-value (one-sided) for statistical significance of t statistic; d, Cohen’s d

Mental Health Problem Domain	Men (N = 129)			Women (N = 54)			Metrics: women > men					
	Better	Worse	Net	Better	Worse	Net	Better		Worse		Net	
							T-test result	Effect size	T-test result	Effect size	T-test result	Effect size
	Mean (SD)	Mean (SD)	Mean (SD)	Mean (SD)	Mean (SD)	Mean (SD)	T, P	d	T, P	d	T, P	d
Depression	2.68 (0.94)	0.289 (0.54)	2.39 (1.26)	3.00 (0.82)	0.304 (0.73)	2.69 (1.23)	2.13, 0.017	0.35	-0.15, 0.558	-0.02	1.48, 0.070	0.24
Suicide	2.59 (0.98)	0.125 (0.27)	2.46 (1.05)	2.84 (0.78)	0.146 (0.34)	2.69 (0.98)	1.65, 0.050	0.27	-0.45, 0.672	-0.07	1.36, 0.087	0.22
Substance abuse	2.64 (0.98)	0.216 (0.44)	2.42 (1.09)	2.92 (0.82)	0.206 (0.60)	2.72 (0.98)	1.88, 0.031	0.31	0.13, 0.450	0.02	1.73, 0.043	0.28
Self-harm	2.63 (1.00)	0.146 (0.28)	2.48 (1.09)	2.92 (0.88)	0.206 (0.52)	2.72 (1.03)	1.90, 0.030	0.31	1.01, 0.844	-0.16	1.36, 0.088	0.22
Social function	2.73 (1.01)	0.299 (0.65)	2.43 (1.32)	3.15 (0.85)	0.228 (0.53)	2.92 (1.09)	2.64, 0.005	0.43	0.71, 0.240	0.11	2.37, 0.009	0.38
Self-esteem	3.11 (0.82)	0.255 (0.59)	2.85 (1.19)	3.30 (0.76)	0.201 (0.49)	3.10 (1.02)	1.52, 0.065	0.25	0.59, 0.278	0.10	1.35, 0.089	0.22
All domains combined	2.75 (0.74)	0.251 (0.38)	2.50 (.93)	3.07 (0.59)	0.229 (0.48)	2.84 (0.79)	2.83, 0.003	0.46	0.33, 0.372	-0.05	2.36, 0.010	0.38

The right side of Table 7 shows the results of one-sided t-tests testing the operational hypotheses that women reported higher positive change (“Better”), lower negative change (“Worse”), and higher net change. With one exception, mean positive change reported by women was significantly higher than that reported by men; the exception, self-esteem, approached significance at p = .065. The difference in reported positive change for all domains combined, with women reporting higher positive change than men, was highly significant, at p = 0.003. Women did not consistently report lower negative change than men, but these differences, and the absolute value of the negative changes reported by both men and women, were so small as to be relatively trivial. No p-value testing sex differences in reported negative change was significant, and all the effect sizes were small. As hypothesis H3 predicted, subtracting negative change from positive change, women reported moderately but significantly higher net psychological change over all domains combined, at 2.8, than did men, at 2.5, with an effect size of 0.38. 

It should also be noted that, despite the differences, both men and women perceived psychological improvement (symptoms getting better) with sexual orientation change to be an order of magnitude greater than perceived psychological worsening (symptoms getting worse). For men, the largest rating for negative change was 0.299, for social function; the largest rating for positive change was for self-esteem, at 3.11, more than 10 times larger. The corresponding comparison for women was 0.304, for negative change with depression, and 3.30, for positive change with self-esteem, also more than 10 times larger. On the other hand, although they were small, all the ratings for negative change were significantly different from zero, by t-test, for both men and women. For all domains, both individually and combined, for both men and women, net change was invariably positive and of relatively large magnitude. Clearly, in the minds of these participants, leaving pride behind was far more psychologically beneficial than harmful.

Association of therapy with harm or benefit

Participation in SRT generally improved psychological outcomes above the positive association with sexual orientation change alone for both men and women, though most effect sizes were small. Table 8 presents the data. The table presents the mean net improvement by SRT exposure over all cases and for men and women separately. T-tests evaluate overlapping hypotheses that net psychological improvement was higher with SRT than without it, and net psychological improvement was higher for women than for men.

**Table 8:** Sex differences in the association of SRT exposure with net psychological improvement after sexual orientation change. T-tests tested the hypotheses that net psychological improvement was higher with SRT than without SRT, and that the SRT effect was higher for women than for men. Cases are reduced due to item nonresponse. SRT, sexual reorientation therapy; net psychological improvement, reported positive change (better) minus reported negative change (worse); N, number of cases; SD, standard deviation; P, p-value (one-sided) for statistical significance of t statistic; d, Cohen’s d

Mental Health Problem Domain	Net psychological improvement						SRT > No SRT						Women > Men			
	Total Cases		Men		Women		Total Cases		Men		Women		No SRT		SRT	
	No SRT (N = 69)	SRT (N = 98)	No SRT (N = 47)	SRT (N = 68)	No SRT (N = 22)	SRT (N = 30)	T-test result	Effect size	T-test result	Effect size	T-test result	Effect size	T-test result	Effect size	T-test result	Effect size
	Mean (SD)	Mean (SD)	Mean (SD)	Mean (SD)	Mean (SD)	Mean (SD)	T, P	d	T, P	d	T, P	d	T, P	d	T, P	d
Depression	2.21 (1.39)	2.69 (1.21)	2.18 (1.33)	2.53 (1.32)	2.27 (1.55)	3.07 (0.79)	2.41, 0.009	0.38	1.39, 0.083	0.26	2.45, 0.009	0.69	.25, .402	0.06	2.09, .020	.46
Suicide	2.36 (1.20)	2.65 (0.97)	2.30 (1.19)	2.56 (1.05)	2.48 (1.24)	2.85 (0.75)	1.73, 0.043	0.27	1.24, 0.109	0.24	1.34, 0.094	0.38	0.58, 0.281	0.15	1.37, .086	.30
Substance abuse	2.52 (1.07)	2.49 (1.13)	2.31 (1.08)	2.48 (1.20)	2.95 (.93)	2.51 (0.98)	0.17, 0.566	0.03	0.74, 0.231	0.14	1.60, 0.942	-0.45	2.35, 0.011	0.61	.15, .442	.03
Self-harm	2.36 (1.15)	2.70 (1.08)	2.21 (1.13)	2.66 (1.15)	2.69 (1.16)	2.80 (0.93)	1.94, 0.027	0.30	2.07, 0.021	0.39	0.40, 0.346	0.11	1.63, 0.054	0.42	.62, .269	.14
Social function	2.42 (1.35)	2.69 (1.31)	2.32 (1.35)	2.48 (1.43)	2.64 (1.34)	3.16 (0.84)	1.28, 0.101	0.20	0.60, 0.275	0.11	1.73, 0.045	0.49	0.90, 0.185	0.23	2.42, .009	.53
Self-esteem	2.93 (1.16)	2.92 (1.23)	2.87 (1.09)	2.82 (1.37)	3.07 (1.31)	3.14 (0.81)	0.06, 0.524	0.01	0.18, 0.571	0.03	0.23, 0.408	0.07	0.66, 0.254	0.17	1.16, .125	.25
All domains combined	2.48 (0.96)	2.70 (0.93)	2.37 (0.92)	2.57 (1.03)	2.71 (1.03)	2.97 (0.56)	1.49, 0.070	0.23	1.10, 0.138	0.21	1.20, 0.117	0.34	1.37, 0.088	0.35	1.99, .025	.44

SRT exposure modestly increased reported improvement in net psychological outcomes over all domains, from 2.48 for those not exposed to SRT to 2.70 for those exposed to SRT, with an effect size of 0.23. This contrast approached statistical significance, at p = 0.07. The results varied by sex and problem domain. Psychological outcomes improved with SRT the most for depression, suicide, and self-harm, with effect sizes from 0.27 to 0.38, all of which were statistically significant. Psychological outcomes improved the least with SRT for self-esteem, substance abuse, and social function, with effect sizes from 0.01 to 0.20, none of which were statistically significant. The sex difference favoring women was strongest for depression and social function, with statistically significant differences for both SRT exposure and sex. The sex difference favoring women was also stronger among persons exposed to SRT than for those not exposed to SRT for all domains except substance abuse and self-harm, where the pattern was reversed.

These results thus support hypothesis H3, which predicted that women would benefit psychologically from SRT more than men, but the effect is small. Over all domains, the net psychological benefit with SRT for women (2.97) exceeded that for men (2.57) by 0.40 points, with an effect size of 0.44, an excess only slightly larger than the amount (0.34) by which women’s net psychological benefit without SRT exceeded that of comparable men (2.71 over 2.37), with an effect size of 0.35. The difference with SRT was statistically significant, at p = 0.025. As already noted, substance abuse and self-harm were exceptions to this rule. The effect of SRT on female substance abuse appeared to be highly negative (effect size -0.45) due to the very high estimated net improvement among women without SRT (2.95), although there was little sex difference in net improvement in substance abuse with SRT (2.48 for men, 2.51 for women).

Association of therapy with suicide ideation

The effect of SRT exposure on suicidality is a perennial concern in the literature and merits further investigation. Consistent with most LGBT research, suicidality was highly prevalent in this population. Prior suicide ideation was reported by 46.7% (SD 50.0) of respondents, comprising 41.7% (SD 49.5) of men and 58.0% (SD 49.9) of women (p = 0.055).

To examine more closely the association of therapy with suicidality, Table 9 overlays the reported net improvement in suicide symptoms with respondents’ history of suicide ideation. The first column (labeled “All”) reports overall net improvement, repeating for “Any SRT” values already reported in Table 8. The second and third columns elaborate on those findings by the presence or absence of previous suicidal ideation. Inspection of the “No therapy” row reveals that, for the members of this sample who were not exposed to any type of therapy, the net benefit for suicide symptoms was higher for respondents who did not report any prior suicide ideation.

**Table 9:** Reported net improvement in suicide symptoms with therapy, by suicide ideation history Therapy types were not mutually exclusive; respondents could report participating in multiple types of therapy. Case counts may be reduced due to item nonresponse. n, number of cases; SD, standard deviation; T, t statistic; P, p-value (one-sided) for statistical significance of t statistic; d, Cohen’s d; SRT, sexual reorientation therapy

Therapy exposure	Total cases	Prior suicide ideation?		No = Yes		Effect size
		No (n = 88)	Yes (n = 77)			
	Mean SD	Mean SD	Mean SD	T	P	d
Men only						
No therapy (n = 30)	2.32 (0.89)	2.38 (0.61)	2.17 (1.55)	0.53	0.572	0.23
Any SRT (n = 68)	2.56 (1.05)	2.31 (0.89)	2.83 (1.15)	2.08	0.021	0.50
Change SRT (n = 56)	2.60 (1.04)	2.38 (0.83)	2.81 (1.19)	1.57	0.061	0.42
Non-change SRT (n = 39)	2.50 (1.18)	2.08 (1.16)	2.89 (1.09)	2.24	0.016	0.72
Non-SRT therapy (n = 41)	2.46 (1.34)	2.00 (0.29)	2.95 (0.27)	2.40	0.011	0.75
Women only						
No therapy (n = 6)	2.08 (2.08)	2.53 (0)	1.85 (2.64)	0.34	0.626	0.30
Any SRT (n = 30)	2.85 (0.75)	2.46 (0.49)	3.24 (0.79)	3.07	0.003	1.16
Change SRT (n = 15)	2.79 (0.70)	2.56 (0.28)	3.14 (0.72)	1.60	0.068	0.86
Non-change SRT (n = 17)	2.82 (0.82)	2.30 (0.73)	3.08 (0.81)	1.84	0.043	0.99
Non-SRT therapy (n = 31)	2.77 (0.86)	2.23 (0.84)	3.18 (0.70)	3.33	0.001	1.26
Total cases						
No therapy (n = 36)	2.29 (1.13)	2.38 (0.13)	1.95 (0.70)	0.81	0.790	0.29
Any SRT (n = 98)	2.65 (0.97)	2.35 (0.80)	2.96 (1.06)	3.15	0.001	0.64
Change SRT (n = 71)	2.64 (0.98)	2.40 (0.14)	2.89 (0.22)	1.96	0.027	0.47
Non-change SRT (n = 56)	2.59 (1.09)	2.13 (1.08)	2.96 (0.99)	2.97	0.002	0.81
Non-SRT therapy (n = 72)	2.59 (1.16)	2.08 (1.15)	3.05 (1.00)	3.77	0.000	0.90

Therapy exposure reversed that association, indicating that those who had seriously considered suicide reported a larger benefit from the therapy than those who had not. Over all cases, therapy was associated with an increase of about three points, ranging from 2.89 to 3.05, in previously suicidal ideating respondents’ five-point rating of positive net change in suicidality, compared to less than 2.5 points, ranging from 2.08 to 2.40, for those not reporting previous suicidal ideation. This effect was somewhat smaller for men than for women, as indicated by the effect sizes involved. The targeted beneficial effect of any SRT for previously suicidal women, at 1.16, was more than twice that for previously suicidal men, at 0.50. Respondents without a history of suicide ideation reported smaller increases in benefit from therapy for suicidality, with an overall effect size of 0.30, which varied little by sex.

The increased benefit of SRT for respondents with past suicidal ideation was statistically significant for both men and women, with an effect size for women (d = 1.16) over twice as large as that for men (d = 0.50). The effect and corresponding sex difference was smaller for change SRT than for non-change SRT, for both men and women, though largely because the benefit of change SRT for those without past suicidal ideation was higher than the benefit of non-change SRT. Non-SRT therapy, which may have addressed psychological issues related to suicide more directly, had a slightly higher observed positive effect on previously suicidal persons in the sample than did non-change SRT.

As already noted, exposure to SRT was associated with reported improvement in suicidal symptoms for both men and women, though more strongly for women. The sensitivity analysis of this section demonstrates that the perceived psychological benefit of SRT on suicidality was higher for persons who previously were more suicidal. The association of SRT and therapy generally, with greater suicidality benefit among those troubled by suicide ideation, provides additional evidence confirming that SRT, like other forms of therapy, was positively associated with reported improvement, not harm, and was more helpful to the respondents more in need of help.

## Discussion

This study presents the fourth independent sample of persons reporting a homosexual to heterosexual orientation change who report similar substantial reductions of SSA and SSB with psychological benefit, not harm. Spitzer [19] found that about two-thirds of participants experienced an incremental movement toward heterosexuality in SSA, with 99% of men and 100% of women reporting complete elimination of SSB. Karten and Wade [8] reported “a statistically significant decrease in reported homosexual feelings and behavior” of 35%, with improved psychological function, among 117 men undergoing SRT. They did not report results for SSA and SSB separately. Sullins and Rosik [12], examining reports from 125 male SRT recipients, also found that the majority reported incremental mean change toward heterosexuality in attractions and behavior. A reanalysis of this sample using similar measures to those of the present paper found that 100% of the sample experienced an LSSB shift [11]. These disparate prior studies share the following characteristics with the present study: 1) Persons successfully undergoing change were highly religious, seeking to reconcile SSA with core religious beliefs that prohibited or problematized homosexual behavior. 2) When the dimensions of sexual orientation were measured separately, change was usually partial and incomplete for SSA but was strikingly complete and total for SSB. 3) Following the change, most people no longer identified as LGB, although they did not necessarily identify as heterosexual. 4) The effect of change on reported mental health was overwhelmingly beneficial, not harmful. At least two other studies have also presented similar findings using less comparable, albeit in some ways methodologically stronger, measures and methods [16,18].

Unlike previous studies of this population based on clinical samples, the present study was able to distinguish the psychological effects of the change transition itself from those of the therapy that may have supported the transition. From this analysis, I found that therapy reportedly added little to the strong, positive psychological effect of the transition journey itself. For every psychological domain measured, and for both men and women, those reporting much improvement in psychological symptoms associated with their personal change journey outnumbered those reporting much worsening of symptoms by over 10 to 1. The improvement in symptoms with SRT was modest compared to the much stronger positive psychological outcomes reported with sexual orientation change itself. In an issue of particular concern for this population, SRT also improved suicidal ideation outcomes much more strongly for those with a history of such morbidity, in strong and statistically significant effects for both men and women. Whether due to therapy or to the transition that therapy may have helped facilitate, however, almost all respondents reported strong, positive psychological benefits associated with their change journey.

Sex differences in sexual orientation change

The change journey of women was found to be different from that of men in several important respects. For women, changing sexual orientation involved greater changes in SSA and SSB, which they were able to make more readily than men. In the process of change, women received much more assistance from SRT and reported more psychological benefits following it.

As reported above, although women were significantly more likely than men, at 85% to 66%, to have received therapy at all during their change journey, men and women were about equally likely to have been exposed to some form of SRT, at 53% to 56%. Men were significantly more likely than women, at 49% to 29%, to have engaged in therapy specifically to help change attractions, behavior or identity, but women were much more likely (at 57% to 32%, t = 3.31, p = 0.001, effect size d = 0.54) to have received non-SRT therapy for other psychological conditions not directly related to sexual orientation change.

Consistent with the present findings, the population representative Generations data of LGB persons also recorded a significantly smaller proportion of women than men (5.8% to 8.6%, t-test p = .031, effect size d = 11.1) who had undergone change therapy. Adjusting for the difference in the proportions of men and women between the two sets of data, i.e., women comprise 52.7% of the Generations sample but only 29.5% of the present study’s LPB sample, yields very similar sex compositions of change therapy participants. In the Generations data, the group exposed to change therapy was 58.0% male and 42.0% female [2]; in the present data, they were 59.6% male and 40.4% female. This close similarity offers criterion validation of the accuracy of the present findings compared to the representative sample.

For both men and women, albeit more sharply for men, change in sexual attraction was very different than change in sexual behavior. On the one hand, the reported effects of therapy on SSA change were sharply differentiated by sex. All forms of therapy had a strong positive association with women’s ability to change from high to low levels of SSA. All or almost all women (93% to 100%) who had engaged in therapy had undergone such an LSSA shift, compared to only 60% of women who had not undergone any therapy. For men’s prospect of changing SSA, therapy had an unforeseen effect: therapy did not merely fail to promote SSA change but was negatively associated with making an LSSA shift. Consistent with this finding, women were also more likely to experience SSA change than men, regardless of therapy participation. Almost all women (88%), but less than half of men (39%), underwent an LSSA shift (Table 4). These results thus strongly confirmed hypothesis H1 (that sexual orientation change and SRT will be associated with a greater reduction in SSA for women than for men). This finding is consistent with past research, which has often found women’s sexuality and sexual orientation dispositions to be more fluid and flexible than those of men. [6].

An alternative interpretation of the negative association of SRT with SSA change for men is that men for whom reducing SSA was more difficult were also more likely to seek therapy support for attempting to reduce SSA. In support of this explanation is the fact that a much higher proportion of women (94%) than men (39%) overall made an LSSA shift, suggesting that men’s SSA was less susceptible to change by any means. Moreover, the proportions of men and women making an LSSA shift in the absence of therapy are relatively similar (55% of men and 60% of women) compared to the proportions doing so with therapy (47% of men and 98% of women). SSA change for men may have been more strongly associated with other efforts, such as individual religious study, retreats, a support group, or a mentoring relationship, which were not examined in this study. These considerations suggest that whatever other effects may be operative, women’s high proportion of LSSA success was related to their positive engagement with therapy, while men’s lower proportion of LSSA success was related to their negative engagement with therapy.

On the other hand, there was virtually no difference between men and women regarding the change in SSB, which all survey respondents experienced in the extreme. Strikingly, by the metric of a definite shift from high SSB to low SSB, 100% of both men and women reported making such an LSSB (“low same-sex behavior”) shift (Table 4). A higher proportion of women than men (56% to 34%; t-test p = 0.009) engaged in more frequent SSB prior to change, so women could be considered to have changed SSB more; however, both men and women changed SSB to the greatest extent possible, so the difference is moot. Due to this unexpectedly extreme result, for which there was no sex variation, it was not possible in these data to test hypothesis H2 (that sexual orientation change and SRT will be associated with a greater reduction in reported SSB for women than for men). However, this was because the therapy was universally successful: all people who may have undergone therapy related to SSB change attained that goal.

Consistent with every previous study of persons reporting successful sexual orientation change, doing so was reportedly much more psychologically beneficial than harmful, in this study by a ratio of over 10 to 1, as Table 7 reports. As with SSA and SSB reduction, the increase in psychological well-being was more strongly associated with sexual orientation change than with SRT. Undergoing SRT to help change sexual orientation added only a relatively small amount to the much larger reported net psychological benefit of changing sexual orientation itself, with or without SRT. Both sets of psychological improvement-that from changing sexual orientation and that from SRT-were larger for women than for men. These results thus supported hypothesis H3 (that women will report more positive net psychological outcomes associated with sexual orientation change and with SRT).

Of course, improvement in psychological distress can only occur among those experiencing distress, or sufficient distress, to begin with, suggesting the average measures of psychological outcomes can be misleading. In this respect, the sensitivity analysis of the effect of SRT on suicide ideation helpfully confirmed that the reported effect of SRT was stronger for persons with higher prior suicide ideation. SRT was most helpful for persons most in need of help. 

The positive effect of SRT on suicidality in this study contrasts sharply with the repeated claim that SRT increases suicidality. As I have already suggested, the difference is an artifact of different sample restrictions. The stark difference between the strong positive psychological outcomes observed for samples restricted to formerly LGBT but currently non-LGBT persons who have undergone sexual orientation change and the strong negative outcomes observed for samples restricted to currently LGBT persons who have not undergone sexual orientation change underlines the fact that the true effect of SRT on those undergoing this form of therapy can only be estimated from information on both sets of outcomes, which is to say, from the set of all persons exposed to this therapy.

The present data, which contained 28 LGB cases, strongly confirm that persons who have successfully reduced SSA or SSB are also more likely to have desisted from an LGB identification. The overall reported mean SSA change of -2.83 (Table 2) was partitioned into -1.22 (SD 1.22) for LGB respondents but -3.14 (SD 1.90) for all other, mostly heterosexual, respondents. This contrast was statistically significant by t-test at 0.0001, and was similar, and also statistically significant, for both men and women. SSB reduction was -1.5 (SD 1.37) for LGB persons but -2.74 (SD 2.01) for all other persons (difference p = 0.002), with a similar difference among men only but not among women only. SRT participation was associated with reduced psychological improvement for LGB identified persons (from 2.60 to 2.07, p = 0.341) but with increased improvement for persons of all other identifications (from 2.40 to 2.83, p = 0.056) in the sample. Clearly, if the present study had been restricted to current LGB-identified cases, it would have starkly understated the effectiveness and psychological benefit of SRT for sexual orientation change.

It is thus not possible to conclude that SRT is universally ineffective and harmful while excluding non-LGBT SRT alumni any more than it is possible to conclude that SRT is mostly effective and harmless while excluding LGBT alumni. The true effect of SRT on those undergoing this form of therapy can only be estimated from information on both sets of outcomes, which is to say, from the set of all persons exposed to this therapy. The present findings suggest, moreover, that most of the difference in outcomes between these two populations derives not from SRT but from the experience of changing sexual orientation itself, regardless of therapy exposure. 

Replicating previous findings: the Spitzer study

The sex differences in sexual orientation change elements found in this study are very similar to those reported in Dr. Robert Spitzer’s landmark 2003 study of sexual orientation change [19]. Spitzer, an eminent psychiatrist who had been influential in removing homosexuality from the American Psychiatric Association’s manual of disorders, maintained that while those comfortable with a homosexual condition should not be stigmatized, persons distressed by it should be supported in seeking to change it or manage their discomfort. Spitzer reported on a sample of 143 males and 47 females “who reported at least some minimal change from homosexual to heterosexual orientation,” much like the present study’s sample of 129 males and 59 females who reported homosexual to heterosexual change in at least one dimension of sexual orientation. Although Spitzer’s focus of interest was on change in SSA, he noted that “because sexuality in gay men and lesbians may be experienced and expressed differently, as is the case with heterosexual individuals, gender differences in the reported changes are also examined” [19] (page 405).

Adjusting when needed for differences in measurement scales, the findings of the present study regarding sex differences in SSA and SSB change are similar in many respects to the results of Spitzer’s study. The following comparison statements present values from Spitzer’s study, followed by the corresponding value from the present study in parentheses. Parenthetical page numbers reference Spitzer’s study. The present study values shown are restricted to therapy participants only, as was Spitzer’s data.

Regarding SSA, the two studies found that over the change transition, the proportion of males reporting high SSA decreased by 47% (51.7%), well below the corresponding proportion of females, which dropped by 84% (93.5%) (page 411). Prior to transition, 46% (51.2%) of males and 42% (45.3%) of females reported experiencing SSA exclusively, with no OSA (page 409). After transition, 17% (14.3%) of males and 54% (37.8%) of females reported exclusive OSA, with no SSA (page 409). Complete absence of SSA after transition was reported by 11% (14.1%) of males and 37% (56.5%) of females (page 410).

Regarding SSB, prior to transition, 26% (11%) of men and 33% (11%) of women reported never engaging in homosexual sex, and one percent (5%) of men and 5% (15%) of women reported doing so daily or almost daily. The differences in this comparison may reflect the fact that Spitzer’s study was based on telephone interviews, a method known to suppress reports of sensitive behavior compared to the anonymous computer-mediated survey method employed in the present study. Both studies agree that after transition, 99% (100%) of males and 100% (100%) of females reported not engaging in any homosexual sex. Spitzer concluded that, in general, “[f]emale participants reported significantly more change than did male participants” (page 403), a summary statement that also applies to the present study.

Spitzer reported only one psychological effect: “Depression has been reported to be a common side effect of unsuccessful attempts to change sexual orientation. This was not the case for our participants, who often reported that they were “markedly” or “extremely” depressed at PRE [prior to change] (males 43%, females 47%), but rarely that depressed at POST [following change] (males 1%, females 4%). To the contrary, at POST, the vast majority reported that they were “not at all” or only “slightly” depressed (males 91%, females 88%) (page 412). The present study’s positive and negative psychological change variables present a contrast very similar to that of Spitzer’s PRE and POST measures of depression. Therapy participants exposed to depression often reported “extremely much” or “very much” positive change in their condition (males 58.8%, females 86.1%) but rarely reported that much negative change (males 3.0%, female 5.6%). The common finding of both studies is that SRT was not associated with increased depression, but rather greatly reduced depression, for those who were successful in changing sexual orientation.

Denying change

The repeated finding of self-reported sexual orientation change in the studies reporting successful change cited above (“positive SRT studies”) is consistent with already-noted genomic evidence that sexual orientation is not an immutable genetic trait and population evidence that it often changes over the life course [20,27]. Despite this converging evidence, reports of homosexual to heterosexual change are widely rejected by psychological scholars on speculation that those making them were “especially susceptible to believing and reporting that therapy has succeeded regardless of its true effectiveness” [21]. Such suspicions are often accompanied by a statement of the author’s belief that sexual orientation cannot change, and are offered, in all cases I know, with no supporting evidence. Similar suspicions of false report or belief have also never been applied to similar self-reports of heterosexual to homosexual change (“coming out”), which are readily accepted at face value. Dr. Spitzer, for example, later questioned the credibility of his 2003 study of homosexual to heterosexual change after activist pressure, prompting a protest from a group of sample members at having their honesty impugned [28]. No similar concern has ever been raised regarding the dozens of studies of reports of heterosexual to homosexual change. As noted above, findings on sexual orientation change are sharply different between non-LGBT samples, such as Spitzer’s and the present one, which report moderate change, and samples restricted to LGBT persons, who report little or no change. To my knowledge, no critic has yet addressed why the latter should be believed but the former should not. It is difficult, therefore, to discount the probability that the uniquely suspicious reception of contrary findings may express on this topic the well-documented scholarly bias among psychologists [29] in the form of special pleading.

This debate may also reflect different understandings of sexual orientation by each side. If therapy success is defined as changing internal attractions, as psychologists tend to do, then it is possible to argue that not much change occurred in the positive SRT studies, particularly for men. Despite the suspicions of fabulism, most men in these studies do not report large degrees of SSA change. The positive SRT studies, including the present study, have found that, while SSB reduction was complete and total, not all men were able to reduce SSA; most men did not reduce SSA completely, and it is possible that few, if any, men eliminated SSA entirely. In the present study, only five men (4%) reported SSA change from the highest (“extremely much”) to the lowest category (“none”), while over three times that many (14%) who began in the highest SSA category reported no change at all (See Figure 1). Similar contrasts have been reported for other SRT samples [11,18]. This is not a distribution that supports a claim of large-scale distortion or lying by persons determined to show change to be successful. On the contrary, it suggests that most participants acknowledged internal attractions that were resistant to change. It is plausible that some or all of the 4% reporting complete change may have misunderstood the question, inadvertently misstated their reduction in SSA, or made other errors. This small proportion is well within the range of such indeterminate or outlier responses on any self-report survey. Those who claim that this population is uniquely susceptible to overstating homosexual to heterosexual SSA change must explain why, if that were true, three times more men reported no change than reported complete change. 

The present study strengthens this point by showing that men undergoing SRT reported less LSSA shift than those not undergoing SRT, a contrast that was strongest for therapy focused on SSA change. This finding explicitly contradicts the dismissive speculation that men undergoing SRT were unusually likely to overstate the success of therapy. It also, of course, confirms the possibility that SRT is less effective for men than other interventions or efforts to reduce SSA, but only if the men in the sample are accepted as reasonably honest reporters, on par with most other self-report samples.

Beyond this consideration, the thesis that SRT cannot be effective because sexual attractions cannot change founders on gender differences. To the extent that the evidence of this study is consistent with claims that sexual attractions are difficult or impossible to change and therapy is not helpful for men to do so, the same evidence precludes such conclusions regarding women. The ratio of no change to the most extreme change among those beginning with the highest SSA, 14% to 4% for men, was reversed, 2% to 19%, for women (see Figure 3). Women’s chances for SSA change greatly improved with SRT, to almost 100%, with statistical significance and a very large effect size. The same contrasts that may suggest that men do not easily change SSA, especially with SRT, also suggest that women change SSA readily, and even more so with SRT.

Competing definitions

A more fundamental theoretical challenge to the idea that sexual attraction determines orientation is presented by the striking finding that, whether or not they shifted from high to low or no SSA, 100% of the men (and women) in the data reported shifting to low or no SSB. The consensus definition of sexual orientation conceives it to be composed of three elements: attraction, behavior, and identity. In these data, all the initially same-sex oriented men who may not have completely left behind the first element nevertheless did completely leave behind the second element and, in most cases, the third element. Whether or not one agrees that they thereby changed sexual orientation, then, depends on whether or not one accepts the view that sexual orientation is comprised of all three elements or, on the contrary, consists merely of attractions. The idea that they failed in changing sexual orientation or that their therapy was not successful because they may still retain some level of SSA attempts to collapse the diversity of sexual experience into only a set of internal dispositions.

The corresponding claim that sexual behavior that does not sufficiently align with attractions is inauthentic, not “who they are,” dismisses both the manifest nature and diversity of sexual expression and the very real religious commitments of these persons. Sex between humans is clearly an interpersonal activity, not merely a personal desire. In the belief systems subscribed to by these highly religious persons, sexual activity, like all human behavior, is not determined by desire but by the design of a creator God. Common to Judaism, Christianity, and Islam is an origin story of the toxic fall from a garden of perfection following the failure to resist the temptation to transgress God’s order. For these religions, the fundamental difference between humans and animals is that the former are able to resist desire on the basis of principles apprehended by reason. Core personal fulfillment comes from accepting or submitting to God’s design, not from expressing personal desire or will contrary to that design. Thus, by resisting SSA to cease SSB, the men in these data were not denying, but finding and fulfilling who they really are. And all men undergoing therapy were able to achieve this result, which, from their religious point of view, was considered successful.

Misunderstanding or dismissal of this point is reflected by assertions that sexual orientation change requires increased OSB, not merely reduced SSB [21]. Such critiques fail to take seriously the religious motivation presented in the previous paragraph. While religious norms are favorable to legitimate heterosexual sexual activity, they do not require it of anyone. All Abrahamic religions make provision for abstinence from sex relations, some with preferred status for lifelong virginity or celibacy, for those who seek to live in strict accordance with religious norms. The data of this study, which show much larger effect sizes for reducing same-sex orientation than for increasing opposite-sex orientation, with complete cessation of SSB and only a small increase in OSB, are consistent with the perception that reducing SSB was a much more important goal for the members of the sample than was increasing OSB. Combined with the limited adoption of heterosexual identity after change, these results suggest that the goal of sample members, in many cases, may not have been to approximate some idea of “normal” heterosexual behavior, but simply to cease proscribed homosexual behavior. A full exploration of identity and opposite-sex attainment, which is beyond the scope of this study, would be valuable to address these possibilities. 

These results also suggest that the idea of sexual orientation may be overdetermined. In theory, sexual attraction and behavior are conceived as joint experiential components of a coherent orientation to sexual desire and practice. In this study, however, change in sexual attraction was quite different than change in sexual behavior. This was true for both men and women, albeit more sharply for men. These differences challenge the notion that “orientation” means the same thing for women as it does for men, as others have suggested [30]. Consistent with data on the general incongruence of the dimensions of sexual orientation [31], they also suggest that nonheterosexual sexual orientation may not refer to a discrete characteristic of human phenotype, but rather, at best, a loose collection of tendencies which are largely variable and independent.

Legal and clinical implications

These findings also have some possible legal and clinical implications. The strong confirmation in these data that women undergoing SRT experience more change and higher psychological benefit than do men suggests that assessments of therapy outcomes that rely on data comprised entirely or wholly of men, as currently informs attempts to restrict SRT in many professional associations, legislatures, and judiciaries, will inevitably understate the effectiveness and psychological benefit of SRT. SRT restrictions for men should thus be considered separately from restrictions for women. To deny women access to therapy, which is likely to help them resolve sexual orientation conflicts and/or benefit them psychologically, based solely or primarily on evidence of the absence or reduction of such benefits among men, may perpetuate a sad pattern of penalizing women for men’s problems, a prima facie violation of women’s rights to fair and equal treatment.

Clinicians faced with a highly religious client struggling with a sexual orientation issue should be aware that SSA in men is resistant to change, but also that this need not prevent a full resolution of unwanted SSB. The results of this study also suggest that other interventions than therapy may be a better path forward for such a client. The data for this study contain further details about therapy and other interventions, such as age at therapy and type of practitioner, which would reward further clinical study beyond the present scope. 

Limitations

The present study’s findings are subject to several important limitations. The findings rely on retrospective self-report measures, which can be inaccurate due to desirability bias and problems of effective recall, although this problem is often overstated [12]. As with all cross-sectional data, associations cannot determine causation. In particular, reverse causation cannot be excluded. It is possible, for example, that men who had more difficulty reducing SSA by other means chose more often to undergo SRT, rather than that SRT was less effective than the absence of therapy for helping men reduce SSA. Due to the small size of some of the design cells, particularly for women, the corresponding associations observed in the paper should be interpreted with caution and reservation, even when the effect size is large.

The data were not drawn to be statistically representative of any larger population. The sample characteristics, i.e., highly religious heterosexually-identified persons, are consistent with previous samples of persons undergoing change with SRT [13], suggesting that the present findings may tell us something about this unique population, but for the same reason make clear that they cannot be generalized to the LGB-identifying population, which typically has very low religious observance and reports a different set of experiences with SRT.

Many relevant variables present in the data could not be explored within the scope of this study, including sexual identity change, gay affirmative therapy (GAT), adverse child experiences (ACEs), motivation for seeking change, marital status, and age period and duration of therapy. Some research has suggested that males who begin with only SSA and no OSA have different developmental trajectories than men with mixed attractions, a difference that may affect their response to SRT [32]. Future research to address these questions in these or other data would be valuable to better understand the complex nature and development of sexual orientation.

## Conclusions

Among the many ways that men and women are different, this study has found, are differences in the way that they resolve difficulties with SSA when they reportedly change sexual orientation. Although both men and women were equally (and completely) successful in desisting from SSB, women were much more successful in reducing SSA, in using therapy to do so, and in benefiting psychologically from the experience. Ultimately, it is perhaps not surprising that the differences between men and women extend to differences between the way men are attracted to women (or other men) and women are attracted to men (or other women).

Research that neglects the higher changeability and benefits of therapy for women likely understates the full range of effectiveness and psychological benefits of SRT. Restrictions based on such one-sided information risk unfairly deprive women of helpful resources based on their reduced helpfulness for men.

Together, these results contrast sharply with general claims that SSA rarely or never changes, especially by intention, but also emphatically rebut the idea that the presence or absence of SSA necessarily determines SSB. Persons with strong religious principles or experience, it appears, can desist from SSB while retaining substantial SSA and simultaneously experiencing strong psychological benefit. For these persons, sexual behavior may become oriented by their religious principles or beliefs, not by their sexual feelings.

## Appendices

Appendix A: List of acronyms

**Table 10:** List of abbreviations and hypotheses

SOGICE	Sexual orientation and gender identity change efforts
LGBT	Lesbian, gay, bisexual, and transgender
LGB	Lesbian, gay, and bisexual
LB	Lesbian and bisexual
GB	Gay and bisexual
SSA	Same-sex attraction
SSB	Same-sex behavior
OSA	Opposite-sex attraction
OSB	Opposite-sex behavior
LPB	Leaving pride behind
GAT	Gay-affirmative therapy
ACEs	Adverse child experiences
H1	Hypothesis 1: SRT will be associated with stronger change in sexual attraction for women than for men
H2	Hypothesis 2: SRT will be associated with stronger change in sexual behavior for women than for men
H3	Hypothesis 3: SRT will be associated with more positive psychological benefit for women than for men

Appendix B: Leaving Pride Behind Survey Questionnaire

Note: This is a paper (flat file) representation generated from an online survey, which had multiple features that cannot be exactly replicated on paper or in a Word document file.  The questions below are accurate as to question wording and response choice, but do not represent the skip logic and variable attributions of the survey as administered.

The questions are presented without alteration, exactly as they were generated from the online survey by the survey provider (QuestionPro).

For more survey details, please consult the self-documenting electronic survey data file, which is archived at the Harvard Dataverse: Sullins, Paul, 2026, "Leaving Pride Behind: Survey of 183 Persons Reporting Homosexual to Heterosexual Sexual Orientation Change", https://doi.org/10.7910/DVN/YY769U. Data are available under the terms of the Creative Commons Attribution 4.0 International license (CC-BY 4.0).

Survey Questions

Have you ever in your life (check all that apply) ...

1.      experienced sexual attraction to someone of the same sex as yourself?

2.      engaged in sexual behavior with someone of the same sex as yourself?

3.      described your sexual identity as gay, lesbian, bisexual (GLB), or something else other than heterosexual (or straight)?

4.      None of the above applies to me.

Survey Directions: For each item, please indicate the response or responses that come closest to describing you. Some questions ask for very personal information. If you prefer not to answer or no responses match your experience, just skip the question by touching the screen or pressing the Tab key.

Background Characteristics

How old are you (in years)?

1.      18

2.      19

3.      20

4.      21

5.      22

6.      23

7.      24

8.      25

9.      26

10.  27

11.  28

12.  29

13.  30

14.  31

15.  32

16.  33

17.  34

18.  35

19.  36

20.  37

21.  38

22.  39

23.  40

24.  41

25.  42

26.  43

27.  44

28.  45

29.  46

30.  47

31.  48

32.  49

33.  50

34.  51

35.  52

36.  53

37.  54

38.  55

39.  56

40.  57

41.  58

42.  59

43.  60

44.  61

45.  62

46.  63

47.  64

48.  65

49.  66

50.  67

51.  68

52.  69

53.  70-75

54.  76-80

55.  Over age 80

Are you male or female?

1.      Male

2.      Female

3.      Other

Which one or more of the following best describes your race or ethnicity?

1.      White

2.      Black or African American

3.      Hispanic or Latino

4.      Asian

5.      Native American or Alaska Native

6.      Hawaiian or Pacific Islander

What is your highest level of education?

1.      High school

2.      Some college

3.      Trade/vocational/technical

4.      Associates

5.      Bachelors

6.      Masters

7.      Professional

8.      Doctorate

Are you ...

1.      Married, opposite-sex

2.      Married, same-sex

3.      Divorced

4.      Widowed

5.      Separated

6.      Never married

7.      A member of an unmarried couple

How old were you when you got married (most recently, if married more than once)?

1.      Younger than 12

2.      12

3.      13

4.      14

5.      15

6.      16

7.      17

8.      18

9.      19

10.  20

11.  21

12.  22

13.  23

14.  24

15.  25

16.  26

17.  27

18.  28

19.  29

20.  30

21.  31

22.  32

23.  33

24.  34

25.  35

26.  36

27.  37

28.  38

29.  39

30.  40

31.  41

32.  42

33.  43

34.  44

35.  45

36.  46

37.  47

38.  48

39.  49

40.  50

41.  51

42.  52

43.  53

44.  54

45.  55

46.  56

47.  57

48.  58

49.  59

50.  60

51.  61

52.  62

53.  63

54.  64

55.  65

56.  66

57.  67

58.  68

59.  69

60.  70-75

61.  76-80

62.  Over age 80

How old were you when you got divorced?

1.      Younger than 12

2.      12

3.      13

4.      14

5.      15

6.      16

7.      17

8.      18

9.      19

10.  20

11.  21

12.  22

13.  23

14.  24

15.  25

16.  26

17.  27

18.  28

19.  29

20.  30

21.  31

22.  32

23.  33

24.  34

25.  35

26.  36

27.  37

28.  38

29.  39

30.  40

31.  41

32.  42

33.  43

34.  44

35.  45

36.  46

37.  47

38.  48

39.  49

40.  50

41.  51

42.  52

43.  53

44.  54

45.  55

46.  56

47.  57

48.  58

49.  59

50.  60

51.  61

52.  62

53.  63

54.  64

55.  65

56.  66

57.  67

58.  68

59.  69

60.  70-75

61.  76-80

62.  Over age 80

How old were you when you became separated?

1.      Younger than 12

2.      12

3.      13

4.      14

5.      15

6.      16

7.      17

8.      18

9.      19

10.  20

11.  21

12.  22

13.  23

14.  24

15.  25

16.  26

17.  27

18.  28

19.  29

20.  30

21.  31

22.  32

23.  33

24.  34

25.  35

26.  36

27.  37

28.  38

29.  39

30.  40

31.  41

32.  42

33.  43

34.  44

35.  45

36.  46

37.  47

38.  48

39.  49

40.  50

41.  51

42.  52

43.  53

44.  54

45.  55

46.  56

47.  57

48.  58

49.  59

50.  60

51.  61

52.  62

53.  63

54.  64

55.  65

56.  66

57.  67

58.  68

59.  69

60.  70-75

61.  76-80

62.  Over age 80

How old were you when you began living together?

1.      Younger than 12

2.      12

3.      13

4.      14

5.      15

6.      16

7.      17

8.      18

9.      19

10.  20

11.  21

12.  22

13.  23

14.  24

15.  25

16.  26

17.  27

18.  28

19.  29

20.  30

21.  31

22.  32

23.  33

24.  34

25.  35

26.  36

27.  37

28.  38

29.  39

30.  40

31.  41

32.  42

33.  43

34.  44

35.  45

36.  46

37.  47

38.  48

39.  49

40.  50

41.  51

42.  52

43.  53

44.  54

45.  55

46.  56

47.  57

48.  58

49.  59

50.  60

51.  61

52.  62

53.  63

54.  64

55.  65

56.  66

57.  67

58.  68

59.  69

60.  70-75

61.  76-80

62.  Over age 80

How old were you when your spouse died?

1.      Younger than 12

2.      12

3.      13

4.      14

5.      15

6.      16

7.      17

8.      18

9.      19

10.  20

11.  21

12.  22

13.  23

14.  24

15.  25

16.  26

17.  27

18.  28

19.  29

20.  30

21.  31

22.  32

23.  33

24.  34

25.  35

26.  36

27.  37

28.  38

29.  39

30.  40

31.  41

32.  42

33.  43

34.  44

35.  45

36.  46

37.  47

38.  48

39.  49

40.  50

41.  51

42.  52

43.  53

44.  54

45.  55

46.  56

47.  57

48.  58

49.  59

50.  60

51.  61

52.  62

53.  63

54.  64

55.  65

56.  66

57.  67

58.  68

59.  69

60.  70-75

61.  76-80

62.  Over age 80

How many children do you have?

1.      None

2.      1

3.      2

4.      3

5.      4

6.      5 or more

Religiousness

How important is religion in your life?

1.      Very important

2.      Somewhat important

3.      Not too important

4.      Not at all important

Aside from weddings and funerals, about how often do you attend religious services?

1.      Daily

2.      More than once a week

3.      Once a week

4.      Once or twice a month

5.      A few times a year

6.      Seldom

7.      Never

What is your religious affiliation?  Is it Protestant, Catholic, Jewish, some other religion, or no religion?

1.      Protestant

2.      Catholic

3.      Jewish

4.      Other religion

5.      No religion

What specific denomination is that, if any?

1.      Southern Baptist Convention

2.      Other Baptist churches

3.      United Methodist Church

4.      Other Methodist churches

5.      Presbyterian

6.      Episcopal Church

7.      Lutheran

8.      Pentecostal

9.      Mormon

10.  African Methodist Episcopal Church

11.  No denomination or non-denominational church

12.  Other

Do you consider yourself Orthodox, Conservative, Reform, or none of these?

1.      Orthodox

2.      Conservative

3.      Reform

4.      None of these

Sexual Identity

Which of the following best describes your current sexual identity?

1.      Heterosexual (straight)

2.      Gay or lesbian

3.      Bisexual

4.      I describe my sexual identity in some other way.

Have you always described your sexual identity this way?

1.      Yes

2.      No, I have described my sexual identity in one or more different ways in the past.

How old were you when you came to adopt your current sexual identity or self-understanding?

1.      Younger than 12

2.      12

3.      13

4.      14

5.      15

6.      16

7.      17

8.      18

9.      19

10.  20

11.  21

12.  22

13.  23

14.  24

15.  25

16.  26

17.  27

18.  28

19.  29

20.  30

21.  31

22.  32

23.  33

24.  34

25.  35

26.  36

27.  37

28.  38

29.  39

30.  40

31.  41

32.  42

33.  43

34.  44

35.  45

36.  46

37.  47

38.  48

39.  49

40.  50

41.  51

42.  52

43.  53

44.  54

45.  55

46.  56

47.  57

48.  58

49.  59

50.  60

51.  61-65

52.  66-70

53.  Over age 70

Before coming to adopt your current sexual identity, during the time you adopted a non-heterosexual identity, which of the following would have best described you?

1.      Gay or lesbian

2.      Bisexual

3.      I described my sexual identity in some other way.

4.      Other

How old were you when you first came to adopt your former non-heterosexual identity?

1.      Younger than 12

2.      12

3.      13

4.      14

5.      15

6.      16

7.      17

8.      18

9.      19

10.  20

11.  21

12.  22

13.  23

14.  24

15.  25

16.  26

17.  27

18.  28

19.  29

20.  30

21.  31

22.  32

23.  33

24.  34

25.  35

26.  36

27.  37

28.  38

29.  39

30.  40

31.  41

32.  42

33.  43

34.  44

35.  45

36.  46

37.  47

38.  48

39.  49

40.  50

41.  51

42.  52

43.  53

44.  54

45.  55

46.  56

47.  57

48.  58

49.  59

50.  60

51.  61-65

52.  66-70

53.  Over age 70

Before you first adopted a non-heterosexual identity, which of the following would have best described you?

1.      Heterosexual (straight)

2.      Gay or lesbian

3.      Bisexual

4.      I described my sexual identity in some other way.

5.      I was not sure what my sexual identity was.

6.      Other

Sexual Experience

Child sexual abuse: Before age 18, how often did anyone at least 5 years older than you, or an adult . . .

Never

Once

More than once

Ever touch you sexually? Was it ...

❏

❏

❏

Try to make you touch them sexually? Was it ...

❏

❏

❏

Force you to have sexual intercourse? Was it ...

❏

❏

❏

Lifetime sexual experience: With how many different men have you ever had sexual contact? (By "sexual contact," we mean the intentional touching of another person's intimate body parts, such as the genitals, anus, or breasts, either directly or through clothing, with the intent to arouse or gratify sexual desire.)

1.      None

2.      1

3.      2

4.      3-5

5.      6-10

6.      11-25

7.      26-49

8.      50-99

9.      100 or more

How old were you the first time you had sexual contact with a man? (By "sexual contact," we mean the intentional touching of another person's intimate body parts, such as the genitals, anus, or breasts, either directly or through clothing, with the intent to arouse or gratify sexual desire.)

1.      Younger than 12

2.      12

3.      13

4.      14

5.      15

6.      16

7.      17

8.      18

9.      19

10.  20

11.  21

12.  22

13.  23

14.  24

15.  25

16.  26

17.  27

18.  28

19.  29

20.  30

21.  31

22.  32

23.  33

24.  34

25.  35

26.  36

27.  37

28.  38

29.  39

30.  40

31.  41

32.  42

33.  43

34.  44

35.  45

36.  46

37.  47

38.  48

39.  49

40.  50

41.  51

42.  52

43.  53

44.  54

45.  55

46.  56

47.  57

48.  58

49.  59

50.  60

51.  61

52.  62

53.  63

54.  64

55.  65

56.  66

57.  67

58.  68

59.  69

60.  70-75

61.  76-80

62.  Over age 80

How old were you the last time you had sexual contact with a man? (By "sexual contact," we mean the intentional touching of another person's intimate body parts, such as the genitals, anus, or breasts, either directly or through clothing, with the intent to arouse or gratify sexual desire.)

1.      Younger than 12

2.      12

3.      13

4.      14

5.      15

6.      16

7.      17

8.      18

9.      19

10.  20

11.  21

12.  22

13.  23

14.  24

15.  25

16.  26

17.  27

18.  28

19.  29

20.  30

21.  31

22.  32

23.  33

24.  34

25.  35

26.  36

27.  37

28.  38

29.  39

30.  40

31.  41

32.  42

33.  43

34.  44

35.  45

36.  46

37.  47

38.  48

39.  49

40.  50

41.  51

42.  52

43.  53

44.  54

45.  55

46.  56

47.  57

48.  58

49.  59

50.  60

51.  61

52.  62

53.  63

54.  64

55.  65

56.  66

57.  67

58.  68

59.  69

60.  70-75

61.  76-80

62.  Over age 80

Lifetime sexual experience: With how many different women have you ever had sexual contact? (By "sexual contact," we mean the intentional touching of another person's intimate body parts, such as the genitals, anus, or breasts, either directly or through clothing, with the intent to arouse or gratify sexual desire.)

1.      None

2.      1

3.      2

4.      3-5

5.      6-10

6.      11-25

7.      26-49

8.      50-99

9.      100 or more

How old were you the first time you had sexual contact with a woman? (By "sexual contact," we mean the intentional touching of another person's intimate body parts, such as the genitals, anus, or breasts, either directly or through clothing, with the intent to arouse or gratify sexual desire.)

1.      Younger than 12

2.      12

3.      13

4.      14

5.      15

6.      16

7.      17

8.      18

9.      19

10.  20

11.  21

12.  22

13.  23

14.  24

15.  25

16.  26

17.  27

18.  28

19.  29

20.  30

21.  31

22.  32

23.  33

24.  34

25.  35

26.  36

27.  37

28.  38

29.  39

30.  40

31.  41

32.  42

33.  43

34.  44

35.  45

36.  46

37.  47

38.  48

39.  49

40.  50

41.  51

42.  52

43.  53

44.  54

45.  55

46.  56

47.  57

48.  58

49.  59

50.  60

51.  61

52.  62

53.  63

54.  64

55.  65

56.  66

57.  67

58.  68

59.  69

60.  70-75

61.  76-80

62.  Over age 80

How old were you the last time you had sexual contact with a woman? (By "sexual contact," we mean the intentional touching of another person's intimate body parts, such as the genitals, anus, or breasts, either directly or through clothing, with the intent to arouse or gratify sexual desire.)

1.      Younger than 12

2.      12

3.      13

4.      14

5.      15

6.      16

7.      17

8.      18

9.      19

10.  20

11.  21

12.  22

13.  23

14.  24

15.  25

16.  26

17.  27

18.  28

19.  29

20.  30

21.  31

22.  32

23.  33

24.  34

25.  35

26.  36

27.  37

28.  38

29.  39

30.  40

31.  41

32.  42

33.  43

34.  44

35.  45

36.  46

37.  47

38.  48

39.  49

40.  50

41.  51

42.  52

43.  53

44.  54

45.  55

46.  56

47.  57

48.  58

49.  59

50.  60

51.  61

52.  62

53.  63

54.  64

55.  65

56.  66

57.  67

58.  68

59.  69

60.  70-75

61.  76-80

62.  Over age 80

Current sexual experience: During the past year, on average, how often did you have sexual contact with a man? (By "sexual contact," we mean the intentional touching of another person's intimate body parts, such as the genitals, anus, or breasts, either directly or through clothing, with the intent to arouse or gratify sexual desire.)

1.      Never

2.      Less than 1 time a month

3.      1-3 times a month

4.      1 time a week

5.      2-3 times a week

6.      4-6 times a week

7.      Daily

During the past year, with how many different men did you have sexual contact? (By "sexual contact," we mean the intentional touching of another person's intimate body parts, such as the genitals, anus, or breasts, either directly or through clothing, with the intent to arouse or gratify sexual desire.)

1.      None

2.      1

3.      2

4.      3-5

5.      6-10

6.      11-25

7.      26-49

8.      50-99

9.      100 or more

Current sexual experience: During the past year, how often did you have sexual contact with a woman? (By "sexual contact," we mean the intentional touching of another person's intimate body parts, such as the genitals, anus, or breasts, either directly or through clothing, with the intent to arouse or gratify sexual desire.)

1.      Never

2.      Less than 1 time a month

3.      1-3 times a month

4.      1 time a week

5.      2-3 times a week

6.      4-6 times a week

7.      Daily

During the past year, with how many different women did you have sexual contact? (By "sexual contact," we mean the intentional touching of another person's intimate body parts, such as the genitals, anus, or breasts, either directly or through clothing, with the intent to arouse or gratify sexual desire.)

1.      None

2.      1

3.      2

4.      3-5

5.      6-10

6.      11-25

7.      26-49

8.      50-99

9.      100 or more

Now think about the time(s) when you were most involved in same-sex sexual activity.

During your period(s) of greatest same-sex sexual activity, how often did you have sexual contact with a man: (By "sexual contact," we mean the intentional touching of another person's intimate body parts, such as the genitals, anus, or breasts, either directly or through clothing, with the intent to arouse or gratify sexual desire.)

1.      Never

2.      Less than 1 time a month

3.      1-3 times a month

4.      1 time a week

5.      2-3 times a week

6.      4-6 times a week

7.      Daily

During your period(s) of greatest same-sex sexual activity, on average, with how many different men did you have sexual contact in a year: (By "sexual contact," we mean the intentional touching of another person's intimate body parts, such as the genitals, anus, or breasts, either directly or through clothing, with the intent to arouse or gratify sexual desire.)

1.      None

2.      1

3.      2

4.      3-5

5.      6-10

6.      11-25

7.      26-49

8.      50-99

9.      100 or more

During the period(s) of greatest same-sex sexual activity, how often did you have sexual contact with a woman: (By "sexual contact," we mean the intentional touching of another person's intimate body parts, such as the genitals, anus, or breasts, either directly or through clothing, with the intent to arouse or gratify sexual desire.)

1.      Never

2.      Less than 1 time a month

3.      1-3 times a month

4.      1 time a week

5.      2-3 times a week

6.      4-6 times a week

7.      Daily

During your period(s) of greatest same-sex sexual activity, on average, with how many different women did you have sexual contact in a year: (By "sexual contact," we mean the intentional touching of another person's intimate body parts, such as the genitals, anus, or breasts, either directly or through clothing, with the intent to arouse or gratify sexual desire.)

1.      None

2.      1

3.      2

4.      3-5

5.      6-10

6.      11-25

7.      26-49

8.      50-99

9.      100 or more

Sexual Attraction

In your entire life, the most you were ever sexually attracted to a woman was:

1.          Not at all sexually attracted

2.      Slightly sexually attracted

3.      Mildly sexually attracted

4.      Moderately sexually attracted

5.      Significantly sexually attracted

6.      Very sexually attracted

7.      Extremely sexually attracted

During the past year, the most you were sexually attracted to a woman was:

1.       Not at all sexually attracted

2.      Slightly sexually attracted

3.      Mildly sexually attracted

4.      Moderately sexually attracted

5.      Significantly sexually attracted

6.      Very sexually attracted

7.      Extremely sexually attracted

During the period(s) when you experienced the greatest same-sex attraction, the most you were sexually attracted to a woman was:

1.       Not at all sexually attracted

2.      Slightly sexually attracted

3.      Mildly sexually attracted

4.      Moderately sexually attracted

5.      Significantly sexually attracted

6.      Very sexually attracted

7.      Extremely sexually attracted

In your entire life, the most you were ever sexually attracted to a man was:

1.        Not at all sexually attracted

2.      Slightly sexually attracted

3.      Mildly sexually attracted

4.      Moderately sexually attracted

5.      Significantly sexually attracted

6.      Very sexually attracted

7.      Extremely sexually attracted

During the past year, the most you were sexually attracted to a man was:

1.        Not at all sexually attracted

2.      Slightly sexually attracted

3.      Mildly sexually attracted

4.      Moderately sexually attracted

5.      Significantly sexually attracted

6.      Very sexually attracted

7.      Extremely sexually attracted

During the period(s) when you experienced the greatest same-sex attraction, the most you were sexually attracted to a man was:

1.      Not at all sexually attracted

2.      Slightly sexually attracted

3.      Mildly sexually attracted

4.      Moderately sexually attracted

5.      Significantly sexually attracted

6.      Very sexually attracted

7.      Extremely sexually attracted

Your Change Journey

How important were each of the following for your motivation to journey away from homosexuality to/toward your current self-understanding?

Not at all important

Slightly important

Moderately important

Very important

Extremely important

Belief that I could only be happy if I overcame my homosexuality.

❏

❏

❏

❏

❏

Belief that homosexuality is unnatural

❏

❏

❏

❏

❏

Conflict between my religion (God) and homosexuality

❏

❏

❏

❏

❏

Desire to be part of mainstream heterosexual society

❏

❏

❏

❏

❏

Desire to be married or stay married

❏

❏

❏

❏

❏

Desire to have my own children

❏

❏

❏

❏

❏

Belief that the gay lifestyle is not emotionally satisfying

❏

❏

❏

❏

❏

My homosexual relationships were emotionally painful

❏

❏

❏

❏

❏

Fear of disease

❏

❏

❏

❏

❏

Disapproval of homosexuality by my parents or siblings

❏

❏

❏

❏

❏

Other (specify after rating)

❏

❏

❏

❏

❏

At this point in your journey, how much change from homosexual life to opposite-sex life would you honestly say you have experienced in your . . .

Complete change

Very much change

Moderate change

Slight change

No change at all

I was always heterosexual and have not changed

Sexual behavior

❏

❏

❏

❏

❏

❏

Sexual identity

❏

❏

❏

❏

❏

❏

Sexual attractions

❏

❏

❏

❏

❏

❏

Counseling Experience

Have you ever seen a professional counselor or therapist (check all that apply) ...

1.      to help you change your sexual identity away from homosexuality and/or to/toward heterosexuality?

2.      to help you reduce same-sex behavior and/or increase opposite-sex behavior?

3.      to help you reduce same-sex attractions and/or increase opposite-sex attractions?

4.      to help you remain or become more comfortable with a homosexual sexual orientation?

5.      to address discomfort or struggle with issues related to your sexuality without expecting or excluding change?

6.      For any other reason?

7.      I have never seen a counselor or therapist for any reason.

What kind of counselor or therapist did you see? Please indicate all that apply and how helpful each was to you:   

N/A, Did not use

Not at all helpful

Slightly helpful

Moderately helpful

Very helpful

Extremely helpful

Psychiatrist (medical doctor)

❏

❏

❏

❏

❏

❏

Psychologist

❏

❏

❏

❏

❏

❏

Pastoral or religious-based counselor

❏

❏

❏

❏

❏

❏

Social worker

❏

❏

❏

❏

❏

❏

Mental health, marriage, or family counselor

❏

❏

❏

❏

❏

❏

Other (specify after rating)

❏

❏

❏

❏

❏

❏

How old were you the first time you met or spoke with a counselor?

1.      Younger than 12

2.      12

3.      13

4.      14

5.      15

6.      16

7.      17

8.      18

9.      19

10.  20

11.  21

12.  22

13.  23

14.  24

15.  25

16.  26

17.  27

18.  28

19.  29

20.  30

21.  31

22.  32

23.  33

24.  34

25.  35

26.  36

27.  37

28.  38

29.  39

30.  40

31.  41

32.  42

33.  43

34.  44

35.  45

36.  46

37.  47

38.  48

39.  49

40.  50

41.  51

42.  52

43.  53

44.  54

45.  55

46.  56

47.  57

48.  58

49.  59

50.  60

51.  61

52.  62

53.  63

54.  64

55.  65

56.  66

57.  67

58.  68

59.  69

60.  70-75

61.  76-80

62.  Over age 80

How old were you the last time you met or spoke with a counselor?

1.      Younger than 12

2.      12

3.      13

4.      14

5.      15

6.      16

7.      17

8.      18

9.      19

10.  20

11.  21

12.  22

13.  23

14.  24

15.  25

16.  26

17.  27

18.  28

19.  29

20.  30

21.  31

22.  32

23.  33

24.  34

25.  35

26.  36

27.  37

28.  38

29.  39

30.  40

31.  41

32.  42

33.  43

34.  44

35.  45

36.  46

37.  47

38.  48

39.  49

40.  50

41.  51

42.  52

43.  53

44.  54

45.  55

46.  56

47.  57

48.  58

49.  59

50.  60

51.  61

52.  62

53.  63

54.  64

55.  65

56.  66

57.  67

58.  68

59.  69

60.  70-75

61.  76-80

62.  Over age 80

Did any of the counseling you received involve painful aversion techniques (painful electric shock, vomit-inducing drugs, beating, etc.)?

1.      Yes

2.      No

3.      Other

Which of the following statements best describes your counseling experience:

1.      Other people forced me to undergo counseling; it was not my free choice.

2.      Although other people did encourage me to undergo counseling, doing so was my own choice.

3.      No one pressured me in any way to undergo counseling; doing so was entirely my own free choice.

4.      Other

Which of the following statements best describes your counseling experience:

1.      The counselor fully supported my goals.

2.      The counselor did not fully support my goals.

3.      Other

Have you ever participated in any activities other than counseling or therapy to address issues related to your sexuality? If so, please indicate any of the following that apply and how helpful each was to you.  (If not, go to the next question.)

N/A, Did not use

Not at all helpful

Slightly helpful

Moderately helpful

Very helpful

Extremely helpful

Weekend or retreat experience

❏

❏

❏

❏

❏

❏

Religious-oriented support group

❏

❏

❏

❏

❏

❏

Non-religious peer support group

❏

❏

❏

❏

❏

❏

Gay-specific educational program

❏

❏

❏

❏

❏

❏

Group intervention or counseling

❏

❏

❏

❏

❏

❏

A mentoring relationship

❏

❏

❏

❏

❏

❏

Intense individual study

❏

❏

❏

❏

❏

❏

Other (specify after rating)

❏

❏

❏

❏

❏

❏

Early family and psychological struggles: The next set of questions asks about problem conditions in your early family, experiences with suicide, and current psychological struggles. These are sensitive topics, and some people may feel uncomfortable with these questions. Your answers are valuable to help us better understand and help persons who experience what you have experienced. We hope you will answer as fully as you can. You are free to skip any question if you feel uncomfortable, unsure, or just prefer not to answer.

Have you ever seriously considered attempting suicide?

1.      No

2.      Yes, just once

3.      Yes, more than once

How old were you the first time you considered attempting suicide?

1.      Younger than 12

2.      12

3.      13

4.      14

5.      15

6.      16

7.      17

8.      18

9.      19

10.  20

11.  21

12.  22

13.  23

14.  24

15.  25

16.  26

17.  27

18.  28

19.  29

20.  30

21.  31

22.  32

23.  33

24.  34

25.  35

26.  36

27.  37

28.  38

29.  39

30.  40

31.  41

32.  42

33.  43

34.  44

35.  45

36.  46

37.  47

38.  48

39.  49

40.  50

41.  51

42.  52

43.  53

44.  54

45.  55

46.  56

47.  57

48.  58

49.  59

50.  60

51.  61

52.  62

53.  63

54.  64

55.  65

56.  66

57.  67

58.  68

59.  69

60.  70-75

61.  76-80

62.  Over age 80

How old were you the most recent time you considered attempting suicide?

1.      Younger than 12

2.      12

3.      13

4.      14

5.      15

6.      16

7.      17

8.      18

9.      19

10.  20

11.  21

12.  22

13.  23

14.  24

15.  25

16.  26

17.  27

18.  28

19.  29

20.  30

21.  31

22.  32

23.  33

24.  34

25.  35

26.  36

27.  37

28.  38

29.  39

30.  40

31.  41

32.  42

33.  43

34.  44

35.  45

36.  46

37.  47

38.  48

39.  49

40.  50

41.  51

42.  52

43.  53

44.  54

45.  55

46.  56

47.  57

48.  58

49.  59

50.  60

51.  61

52.  62

53.  63

54.  64

55.  65

56.  66

57.  67

58.  68

59.  69

60.  70-75

61.  76-80

62.  Over age 80

How old were you when you considered attempting suicide?

1.      Younger than 12

2.      12

3.      13

4.      14

5.      15

6.      16

7.      17

8.      18

9.      19

10.  20

11.  21

12.  22

13.  23

14.  24

15.  25

16.  26

17.  27

18.  28

19.  29

20.  30

21.  31

22.  32

23.  33

24.  34

25.  35

26.  36

27.  37

28.  38

29.  39

30.  40

31.  41

32.  42

33.  43

34.  44

35.  45

36.  46

37.  47

38.  48

39.  49

40.  50

41.  51

42.  52

43.  53

44.  54

45.  55

46.  56

47.  57

48.  58

49.  59

50.  60

51.  61

52.  62

53.  63

54.  64

55.  65

56.  66

57.  67

58.  68

59.  69

60.  70-75

61.  76-80

62.  Over age 80

Did you ever actually attempt suicide?

1.      No

2.      Yes, just once

3.      Yes, more than once

How old were you the first time you attempted suicide?

1.      Younger than 12

2.      12

3.      13

4.      14

5.      15

6.      16

7.      17

8.      18

9.      19

10.  20

11.  21

12.  22

13.  23

14.  24

15.  25

16.  26

17.  27

18.  28

19.  29

20.  30

21.  31

22.  32

23.  33

24.  34

25.  35

26.  36

27.  37

28.  38

29.  39

30.  40

31.  41

32.  42

33.  43

34.  44

35.  45

36.  46

37.  47

38.  48

39.  49

40.  50

41.  51

42.  52

43.  53

44.  54

45.  55

46.  56

47.  57

48.  58

49.  59

50.  60

51.  61

52.  62

53.  63

54.  64

55.  65

56.  66

57.  67

58.  68

59.  69

60.  70-75

61.  76-80

62.  Over age 80

How old were you the most recent time you attempted suicide?

1.      Younger than 12

2.      12

3.      13

4.      14

5.      15

6.      16

7.      17

8.      18

9.      19

10.  20

11.  21

12.  22

13.  23

14.  24

15.  25

16.  26

17.  27

18.  28

19.  29

20.  30

21.  31

22.  32

23.  33

24.  34

25.  35

26.  36

27.  37

28.  38

29.  39

30.  40

31.  41

32.  42

33.  43

34.  44

35.  45

36.  46

37.  47

38.  48

39.  49

40.  50

41.  51

42.  52

43.  53

44.  54

45.  55

46.  56

47.  57

48.  58

49.  59

50.  60

51.  61

52.  62

53.  63

54.  64

55.  65

56.  66

57.  67

58.  68

59.  69

60.  70-75

61.  76-80

62.  Over age 80

How old were you when you attempted suicide?

1.      Younger than 12

2.      12

3.      13

4.      14

5.      15

6.      16

7.      17

8.      18

9.      19

10.  20

11.  21

12.  22

13.  23

14.  24

15.  25

16.  26

17.  27

18.  28

19.  29

20.  30

21.  31

22.  32

23.  33

24.  34

25.  35

26.  36

27.  37

28.  38

29.  39

30.  40

31.  41

32.  42

33.  43

34.  44

35.  45

36.  46

37.  47

38.  48

39.  49

40.  50

41.  51

42.  52

43.  53

44.  54

45.  55

46.  56

47.  57

48.  58

49.  59

50.  60

51.  61

52.  62

53.  63

54.  64

55.  65

56.  66

57.  67

58.  68

59.  69

60.  70-75

61.  76-80

62.  Over age 80

During the past 30 days, about how often did you feel ...

All of the time

Most of the time

Some of the time

A little of the time

None of the time

nervous

❏

❏

❏

❏

❏

hopeless

❏

❏

❏

❏

❏

restless or fidgety

❏

❏

❏

❏

❏

so depressed that nothing could cheer you up

❏

❏

❏

❏

❏

that everything was an effort

❏

❏

❏

❏

❏

worthless

❏

❏

❏

❏

❏

Before age 18, did you live with anyone who ...

Yes

No

was depressed, mentally ill, or suicidal?

❏

❏

was a problem drinker or alcoholic?

❏

❏

used illegal street drugs or abused prescription medications?

❏

❏

was sentenced to serve time in a jail, prison, or other correctional facility?

❏

❏

Before you were age 18, were your parents separated or divorced?

1.      Yes

2.      No

3.      Parents not married

Before age 18, how often did a parent or adult in your home ...

Never

Once

More than once

Don't know/Not sure

ever hit, beat, kick, or physically hurt you in any way (not including spanking)? Was it ...

❏

❏

❏

❏

ever swear at you, insult you, or put you down? Was it ...

❏

❏

❏

❏

For how much of your childhood was there an adult in your household who . . .

Never

A little of the time

Some of the time

Most of the time

All of the time

made you feel safe and protected? Was it ...

❏

❏

❏

❏

❏

tried hard to make sure your basic needs were met? Was it ...

❏

❏

❏

❏

❏

Looking back on your personal change journey, how much positive change (symptoms changing for the better) would you say you have experienced in each of the following areas? Mark only one response for each row. 

None

Slight

Moderate

Very much

Extremely much

N/A - Not Applicable

Self-esteem

❏

❏

❏

❏

❏

❏

Depression

❏

❏

❏

❏

❏

❏

Self-harmful behavior

❏

❏

❏

❏

❏

❏

Thoughts/attempts of suicide

❏

❏

❏

❏

❏

❏

Social functioning

❏

❏

❏

❏

❏

❏

Alcohol/substance abuse

❏

❏

❏

❏

❏

❏

Looking back on your personal change journey, how much negative change (symptoms changing to worse rather than better) would you say you have experienced in each of the following areas? Mark only one response for each row. 

None

Slight

Moderate

Very much

Extremely much

N/A - Not Applicable

Self-esteem

❏

❏

❏

❏

❏

❏

Depression

❏

❏

❏

❏

❏

❏

Self-harmful behavior

❏

❏

❏

❏

❏

❏

Thoughts/attempts of suicide

❏

❏

❏

❏

❏

❏

Social functioning

❏

❏

❏

❏

❏

❏

Alcohol/substance abuse

❏

❏

❏

❏

❏

❏

Thinking about the present time and your overall mental health, which includes stress, depression, and problems with emotions, for how many of the past 30 days would you say your mental health was not good?

1.      None

2.      1

3.      2

4.      3

5.      4

6.      5

7.      6

8.      7

9.      8

10.  9

11.  10

12.  11

13.  12

14.  13

15.  14

16.  15

17.  16

18.  17

19.  18

20.  19

21.  20

22.  21

23.  22

24.  23

25.  24

26.  25

27.  26

28.  27

29.  28

30.  29

31.  30

Now thinking about the time(s) in the past when you experienced non-heterosexual orientation and your overall mental health at that time, which includes stress, depression, and problems with emotions, for how many days of the average 30-day period during that past time would you say your mental health was not good?

1.      None

2.      1

3.      2

4.      3

5.      4

6.      5

7.      6

8.      7

9.      8

10.  9

11.  10

12.  11

13.  12

14.  13

15.  14

16.  15

17.  16

18.  17

19.  18

20.  19

21.  20

22.  21

23.  22

24.  23

25.  24

26.  25

27.  26

28.  27

29.  28

30.  29

31.  30

Is there anything else you would like us to know to better understand you and your change journey? Please share briefly in your own words.

Clicking "Done" below will save your responses and complete the survey.
